# Analysis and Experimental Validation of a Piezoelectric Harvester with Enhanced Frequency Bandwidth

**DOI:** 10.3390/ma11071243

**Published:** 2018-07-19

**Authors:** Haim Abramovich, Idan Har-nes

**Affiliations:** Faculty of Aerospace Engineering, Technion, Israel Institute of Technology, Haifa 32000, Israel; idanhn@gmail.com

**Keywords:** piezoelectric harvester, bimorph, natural frequency, generated power, bandwidth, vibrating beam, spring, end mass, series electrical connection, parallel electrical connection

## Abstract

The use of a single bimorph as a harmonic oscillator aimed at harvesting vibrational energy is not effective due to its inherent narrow frequency bandwidth stemming from the need to adjust the natural frequency of the harvester to the platform excitation frequencies. Therefore, the present research focuses on the development, manufacturing, and testing of an advanced system based on three bimorphs, capable of adjusting their natural frequencies using tip end masses, and interconnected by springs, thus enlarging the system’s bandwidth. An analytical model was developed for three bimorphs interconnected by two springs with three end masses. The model can predict the output generated voltage from each bimorph, and then the total output power is measured on a given outside resistor as a function of the material properties, the geometric dimensions of the vibrating beams, the end-masses, and the spring constants. The analytical model was then compared with data in the literature, yielding a good correlation. To further increase the reliability of the model, a test set-up was designed and manufactured that included three bimorphs with three end-masses connected by two springs. The system was excited using a shaker, and the output voltage was measured for each bimorph for various configurations. Then, the analytical model was tuned based on the test results by introducing two factors, the quality and the stiffness factors, and the predictions of the calibrated analytical model were compared with the experimental results, yielding a good correlation. The calibrated analytical model was then used to perform a comprehensive parametric investigation for two and three bimorphs systems, in which the influences of various parameters—like spring constant, mass value, thickness, and width and length of the bimorph and the substrate beam—on the output generated power were investigated. The main conclusion from this parametric investigation was that by correctly choosing the geometric sizes of the cantilevers, the adequate tip end masses, and the ratio between constants of the springs, the frequency bandwidth is expanded yielding a higher harvested power. Typical harvested power of the present designed system can reach up to 20 mW at the first natural frequency and up to 5 mW for the second natural frequency.

## 1. Introduction

Piezoelectric vibration energy harvesting is a technique to accumulate electrical energy from mechanical vibrations. Converting mechanical vibrations from the ambient environment into electrical power enables us to operate remote small electrical consumers such as wireless sensors or low duty cycle radio transmitters.

These types of remote devices might be used to monitor the structural “health” of bridges (SHM), buildings, airplanes, and other large structures. Instead of stretching power lines to the sensors and radio transmitters or using batteries that must be replaced from time to time, self-powering using piezoelectric harvesters makes the whole system simpler, cost-saving, and require minimal maintenance.

Other applications for the piezoelectric harvesters are data monitoring and data transmission of electrical devices buried in pipe lines or remote places. Another application is to supply electrical power to a data logger device located in a closed package for recording the temperature and acceleration during its transportation (for example, by an airplane or a ship).

The sensors and radio transmitters need to be designed for low power consumption, as the power supplied by piezoelectric harvesters is in the range of milliwatts. In [1], a low power wireless consumption sensor and transmitter combined with piezo harvesting element was designed and assembled.

The main problem with ambient vibration energy harvesting is the random characteristics of both the frequencies and their associated amplitudes. A basic harvester is a single bimorph with an end mass. The use of a single cantilever harvester as a harmonic oscillator to harvest vibrational energy is not effective due to its inherent narrow frequency bandwidth stemmed from the need to adjust the natural frequencies of the harvester to the platform excitation frequencies. The main objective of the present research is therefore to extend the bandwidth of the harvesters by employing three bimorphs, each having a tip end mass and interconnected by springs.

Browsing the open literature presents many interesting applications for piezoelectric harvesters with wideband vibration (see for example [2,3,4,5]). The above applications can be divided in a variety of sub-systems, such as

- Variable bimorph stiffness vs. constant bimorph stiffness. Most of the solutions presented in the literature would control the stiffness of the system and the bimorph to change the bimorph natural frequency (for example, the control can be performed by an external applied load). A multi frequency beams array can lead to a system with constant stiffness.
- Autonomous and pre-adjusted systems. Autonomous solutions would sample the frequency input and accordingly adjust the natural frequency of the system to the frequency input. The pre-adjusted solutions can be applied only prior to the vibration itself.
- Linear and nonlinear applied force systems. Nonlinear solutions might use, for example, an axial compressive force on the bimorph leading to its buckling, thus changing its stiffness and its shape. Linear solutions, for example, use a bimorph under axial force (tensile or compression), changing its stiffness.
- Multi bimorphs systems vs. a single bimorph. A multi bimorphs system would possess a large bandwidth, as it is comprised from a variety of bimorphs with various natural frequencies. The natural frequencies of each bimorph can be varied by adjusting the beam’s stiffness and the springs interconnecting the bimorphs. A single bimorph would be much simpler and cheaper but would have only a single natural frequency and thus has a narrow bandwidth.

The relevant literature reveals many interesting harvesters using other kinds of actuation. Ref. [6], for example, presents a magnetic bimorph harvester, having a magnet acting as a tip mass on a vibrating cantilever beam and two stationary magnets (generating attractive and repulsive forces on the tip mass) on both sides of the tip mass. By varying the distance between the stationary magnets and the tip mass magnet, one might change the natural frequencies of the piezoelectric cantilever, enabling the use of the harvesters in various ambient excitations. Experiments in [6] show that, for a basic frequency of 26 Hz, the achieved natural frequency shift was ±6 Hz. The harvested electrical power was in the range of 240–280 μW. An advanced application of the device presented in [6] having an autonomous capability to control the distance between the magnets is presented in [7]. Another magnetic force–based application is proposed in [8], where two bimorphs with permanent magnets are used. This application is similar to the one presented in [6], however, the stationary magnets where replaced by another bimorph having a magnet at its cantilever tip. This application is claimed to generate more power than a single bimorph by changing the distance between the magnets and their orientation (attractive or repulsive forces). Another interesting approach is to use a bi-stable nonlinear harvester, as described in [9]. The harvester consists of a cantilever bimorph with a magnet at its free end and a stationary magnet located in line with the bimorph axis at some distance from the vibrating tip magnet. The two magnets are in a repulsive orientation (north to north), yielding two stable positions. This bi-stable system causes the bouncing of the bimorph between the two stable positions and thus increasing its amplitude and electrical harvested power. Investigating the influence of the distance between the two magnets on the output generated voltage reveals that a change of the distance from 25 to 2.5 mm would increase the generated voltage by a factor of 2. An additional way of obtaining a bi-stable system is to use a buckled bimorph, as presented by [10]. The buckled structure was achieved by an axial force being applied at the end of the bimorph beam, causing its structural buckling. Using this configuration, the generated power of the compressed bimorph was found to be 10 times larger than the unbuckled one. The maximal axial displacement (ΔL) obtained during the presented tests in [10] was 0.7 mm when using a 55 mm beam length. This nonlinear bi-stable application would increase the generated power of a similar linear system. However, one should note that the main issue, which was not addressed in [10], is the relatively large energy needed to be invested in the system to move the bimorph between its bi-stable positions.

A different way of varying the natural frequencies of a bimorph is to apply an axial tensile force on the beam, causing a variation of its stiffness. An interesting configuration is presented in [11], where a screw provides the axial force. Experiments presented in [11] showed that the natural frequency can be changed up to 20% of the original value, leading to a decrease in the electrical power and the quality factor (the damping increases by 50% due to the tension of the harvester). The force needed to operate the beam to its maximal frequency shift was found to be 22.75 N. An alternative configuration is presented by [12], where a beam equipped with two lateral arms aimed at providing axial loading to alter its resonance frequency. One should note that the two arms are part of the beam’s structure and are also made of piezoelectric material such that the production process might be made cheaper. The experimental results presented in [12] showed that the first natural frequency was shifted from 145 to 170 Hz by inducing tensile axial forces and from 145 to 130 Hz by inducing compressive axial forces. The shifts were achieved by applying −20 V up to +20 V on the two arms. One should remember that for a real product, a microcontroller would be used to monitor the ambient excitation frequency and to adjust the harvester’s resonance frequency. A fully automatic system using a piezoelectric actuator that applies axial stress on the piezoelectric harvester is suggested in [13]. The application of −30 V up to +50 V on the piezo actuator might shift the natural frequency of the vibrating beam from 190 to 150 Hz due to the induced compression loads. The system contains a piezo harvester and an actuator element, a charge capacitor, and a microcontroller that controls the voltage supplied to the actuator. It is interesting to note that the microcontroller possesses a learning procedure memory that might reduce the number of samplings needed to analyze the input frequency and determine the voltage to be applied on the harvester. The energy consumption to shift the natural frequency from 190 to 150 Hz is about 266 μJ. To keep the beam stiffness, the system used 8.7 μW (for the wide range of 190 - 150 Hz). One should note that the harvester outlined in [13] has a few shortcomings:

- Part of the energy produced by the harvester is used by the microcontroller and the actuator;
- For an autonomous application, there is a need for a certain time to analyze the input frequency and shift the natural frequency of the beam;
- The application performs well for a pure sinusoidal input; however, it is sensitive to the input frequency.

An alternative way to shift the frequencies accomplished by varying the position of the end mass is described in [14], where a bimorph together with a screw mounted on a fix end mass form the harvester system. The location of the screw was tuned in advance, and the experiments showed a change of bimorph natural frequencies from 130 to 180 Hz. A future improvement could be to automatically tune the position of the screw.

A quite different approach is to make use of a multi frequency beam array as described in [15]. The array is composed of multiple beams with various lengths and tip end masses. Two electrical configurations were tested: electrical output for each single beam and all the beams being electrically connected in parallel. As expected, the parallel connection showed the highest power output 162 μW. Another design can be found in [16], where multi-patches were placed on a rectangular thin plate. This type of harvester displayed a very wide bandwidth and little electrically generated power. To maximize the output power, a comparison between series and parallel connections was performed. It was shown that the parallel connection of the patches has a better output power for low load resistance, while the series connection presented a better power output for a high load resistance. A further improvement of the array concept is a “connected beam array”, a configuration that has several bimorphs connected mostly by springs at the end masses. Some designs, like in [17], have rigid connections between the bimorphs and they might be arranged in a meandering shape. Other designs like in [18,19] have a rigid connection between the bimorph at the end mass. An interesting configuration is presented in [20], where two piezo beams (each being a unimorph) are clamped at one end and connected to the same end mass at the other end. This design uses the mechanical vibrations to excite the two piezo beams, and through a spring, a magnet will also vibrate inside a coil. Thus, the piezoelectric-induced voltage is enhanced by electromagnetically induced voltage, yielding a higher harvested energy and a larger bandwidth.

Similar to what was described in [18], where a stopper was used to increase the output harvested energy, other designs (see for example [21,22]) use an impact technique applied to the piezoelectric layers by the ambient frequency, thus claiming that the frequency sensitivity is decreased. The ball impact harvester presented in [21] is a 6 DOF harvester device designed for human motion energy harvesting. The bimorph impactor developed and presented in [22] used two bimorphs with different end masses (therefore different natural frequencies) and connected by a driven beam. The impact between the two bimorphs and the driven beam increased the output power. The experiments showed a 30–80 Hz bandwidth. One should note that one of the main shortcomings of the impact-based device is its relatively short product lifecycle due to the grinding of the piezo element and the mechanical structure.

All the solutions and designs presented above have some inherent disadvantages that have to be taken into account. These are:

- The applied force on the bimorph should be adjusted in advance;
- Autonomic solutions need time to evaluate the frequency input and use the power accumulated in the bimorph to change the force/distance to the magnets;
- Designs with only one bimorph have a narrow bandwidth. A slight deviation of the beam’s natural frequency from the excitation frequency would cause the output power to drop dramatically;
- The multi-frequency beam array and the rigid connected beam array do no need any adjustments (in advance or automatic). However, they are expensive due to various bimorphs being used and the complex manufacturing process of both the clamping device and the beams’ shapes. Moreover, that kind of designs is not adaptive

A different approach is presented in [23], where two clamped bimorphs are connected at their end by a common hinge device. The angle between the two bimorphs might, in a future work, be changed and thus can control the natural frequency. Connecting the free end of the cantilever bimorph beams by a spring is proposed in [24]. By a proper design of the spring, the end masses and the stiffness of the two beams carrying each a bimorph, a wideband frequency might be achieved. In contrary to the rigid connection or multi frequency beam array designs, the use of a spring as a connector between the end masses of the bimorphs beams enables the tuning of the natural frequency by changing the spring constant. Reference [24] presents an analytic solution accompanied by numerical results for a certain case having a natural frequency of 20 KHz. Those frequencies are not realistic for ambient mechanical vibrations, which are normally below 500 Hz.

A latter publication in the form of a Master of Science thesis [25] based on [24] contains experimental results without comparison to the analytic model. Moreover, the bimorphs end masses amplitudes were not measured. An expansion of the two bimorphs connected by a spring concept is presented in [26], presenting numerical results for 12 bimorphs connected by springs. Reference [26] does not present experimental results, showing only that the harvested power increases when using 12 bimorphs as compared to a single bimorph. Finally, it was proposed in [26] to manufacture a matrix of spring-connected bimorphs having the advantage of enlarging the frequency bandwidth, increasing the output electrical harvested power, and adding more degrees of freedom to control the systems’ natural frequencies. The problem is, of course, the size and cost of system, a topic not discussed in [26]. Another reference [27] proposed a spring-connected beam array. The reference showed some analytic solutions for nine bimorphs connected with springs and showed various types of spring connections and tip masses without performing any parametric investigations.

To complete the review on wide-band harvesters, it is worth to quote some of the following studies in order to provide the reader with a broad picture on this topic. Shahruz [28] presented a method to design the dimensions of multiple beams having end masses at their tips and yielding high performance band-pass filters. The study does not include the piezoelectric contribution and deals only with the frequencies of the beams due to their root excitation. In a follow-up manuscript, Shahruz [29] presents a procedure for the designing mechanical band-pass filters for the system described in [28]. As in [28], the electrical part of the harvester is not considered when designing the band-pass filter. Xue et al. [30] follow the work of Shahruz [28,29] by numerically investigating an array of multiple bimorphs sandwiching carrying cantilever beams without tip beams. Each beam has a different natural frequency due to different height, thus leading to a large band-pass. Electrical connection in series and in parallel were used to connect the various bimorphs, yielding different harvesting behaviors. It turned out that a mixed electrical configuration would lead to the maximization of the harvested power to 140 μW. Another interesting wide-band pass harvester is presented by Qi [31] in his Ph.D. thesis. Using Shahruz’s [28,29] idea, he presented a device comprising of a clamped-clamped beam that is accelerated equally at its clamped ends due to the ambient excitation. The beam supports several small cantilevers, each of which is tuned to a different frequency due to their tip-end masses. The configuration yielded a wide frequency response between 14.5 and 31 Hz. The study also contains a very good literature survey, updated for the year 2011, with additional devices to those quoted in the present survey. Also, it is worth noting the interesting approach being used in the study to solve the electrical part of the topic, which consists of investigating the complex conjugate impedance matching for the piezoelectric harvesters.

Other researchers, like Lien and Shu in three consecutive contributions [32,33,34], address the topic of array oscillators, similar to what had been described above. In [32], the authors suggest the use of equivalent impedance approach to investigate the electrical response of an array of piezoelectric oscillators having distinct energy harvesting circuits. The problems are investigated using the electrical equivalent circuits connected to external interface electronics, like the standard AC/DC and parallel/series synchronized switch harvesting in inductor (SSHI). In their second paper [33], they continued their studies presented in [32], this time addressing the electrical response of a series connection of piezoelectric energy harvesters and its comparison with the parallel case. A model problem was suggested to evaluate the performance of the harvested power under various interface circuits. The main conclusion was that the parallel-SSHI array system exhibited higher power output with moderate bandwidth improvement, while the series-SSHI system delivered a pronounced wideband at the cost of peak harvested power. Finally, their third contribution [34] presents the modeling of a parallel electrical connection of multiple piezoelectric oscillators having respective electrical rectification aimed at boosting power output and exhibiting broadband energy harvesting. The study displays various choices of electronic circuits, with the electrical response being governed by a set of simultaneous nonlinear equations with constraints indicating blocking by the proposed rectifiers.

Al-Ashtari et al. [35] suggested a multi-bimorphs configuration consisting of cantilever beams being sandwiched between two piezoelectric patches and magnetically tuned using tip-end magnets. The authors derived formulas for calculating the power generated by the harvester having an arbitrary number of piezoelectric elements either connected in series or in parallel. It is shown that optimum harvester design must take both the connected load and the operating frequency into account. They found that, to get a bandwidth enhancement, it is essential that individual rectifiers are used for the bimorphs, a similar conclusion to the one that will be presented based on the present research. Their experimental example, having three bimorphs, showed that the power can be increased by about 340% (yielding a max. peak power of 720 μW) or the bandwidth can be increased by about 500% (leading to 30 Hz) compared to one single bimorph, as a function of the tuning strategy adopted during the tests.

Wu and Shu [36] continued the investigations described in [32,33,34] and developed finite element models for designing electrically rectified piezoelectric energy harvesters, as presented in [32,33,34]. After validating their models using the COMSOL code, they found that a significant broadband (about 10 Hz) can be observed for the parallel connection of the oscillators having the parallel-SSHI circuit, yielding approximately 60 μW, or a bandwidth of 10 Hz with a peak power of 60 μW for series connection of the oscillators having serial SSHI circuit. A recent paper by Dechant et al. [37] continued the trend presented above, trying to enhance the bandwidth of vibration energy harvester system by using three piezoelectric cantilevers having tip masses to achieve a frequency tuning for each cantilever. They found that the bandwidth enhancement by mass tuning is limited and requires several bimorphs with close resonance frequencies. Using a simple power-transfer circuit, where several bimorphs with an individual full wave bridge rectifier are connected in parallel, allows one to extract the electrical power close to the theoretical maximum excluding the diode losses. Experiments were performed on two- and three-bimorphs arrays yielding reasonable agreement with the simulations and demonstrating that the power-transfer circuit influences the frequency dependence of the harvested electrical power.

A different approach is presented by Yang et al. [38] by suggesting a two-dimensional piezoelectric harvester having the shape of a frame equipped with piezoelectric patches bonded on it, thus being capable of vibrating in vertical and horizontal directions due to an external excitation. Note that the harvester can capture the vibrational energy from any arbitrary direction in the 2D plane. Numerical, as well as experimental validation, were performed, including the evaluation of the harvester’s performance, using the electric equivalent circuit, yielding promising results.

Finally, an important study by Miller et al. [39] aimed at optimizing both the piezoelectric mechanical harvester and its accompanied electrical circuit components to maximize the harvested power output by finding the appropriate required mechanical and electrical system parameters, is quoted. Their model, yields an upper bound of the output power and the system effectiveness of complete piezoelectric energy harvesting systems and, hence, can serve as a benchmark. Their work presented a system maximal effectiveness of 48% to be achieved at a frequency of 100 Hz for a given generator volume of 3.38 cm^3^.

Based on what had been unveiled in the introductory part of this manuscript, the present proposed harvester, capable of enhancing its frequency bandwidth, which will be next described, formulated, evaluated, and validated, will provide the existing knowledge published in the literature an additional view and approach for this topic.

## 2. The Analytical Model

**Figure 1:**
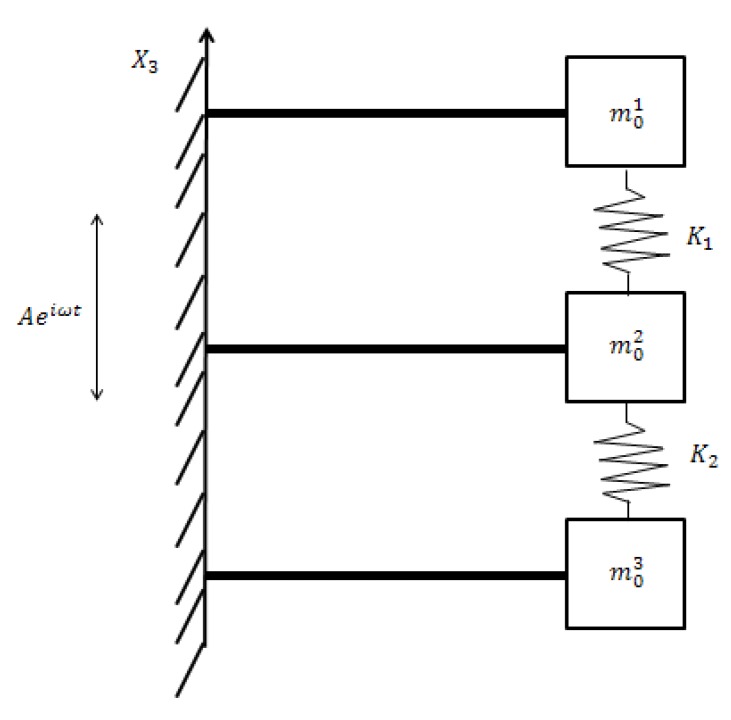
Three bimorphs interconnected by springs—the present harvester design.

Using the basic concept proposed in [24], the present study focuses on an advanced system based on three identical bimorphs bonded on three different cantilever beams with different end masses that are connected by various springs. A schematic drawing of the harvester is presented in Figure 1.

From an electrical point of view, it is known that a series connection between the two piezo strips is used for sensors, while for energy harvesting, the parallel connection is applied. A comparison between series and parallel connections is presented in [16,40,41,42]. In the present study, the parallel connection was also applied (see Appendix B). One should note that the three bimorphs are not electrically connected to each other, and the generated power from each bimorph is separately measured and recorded. The advantages of the proposed concept are:

- The frequency bandwidth is wider than for a single bimorph design;
- Contrary to single bimorph solutions, the present designed system is less sensitive to changes in the input frequency;
- The connection of the bimorphs by springs adds degrees of freedom to the designer by allowing the choice of bimorphs and substrate beams dimensions, end masses sizes, and spring constants;
- Contrary to the multi frequency beam array concept, using the same bimorphs might be cost saving.

The assumptions of the model are:

- Euler-Bernoulli beam theory is applied (see also a discussion about its adequacy in [29,30]);
- A complex damping;
- A perfect adhesion between the metal substrate and the piezo element;
- An ideal clamped beam;
- The end mass connection spring is linear and has the same behavior both in compression and in tension.

**Figure 2:**
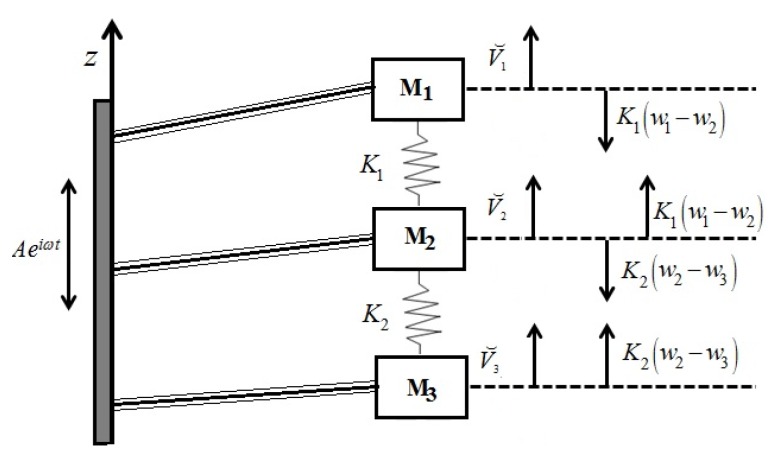
The schematic drawing of the model—the analytic solution.

Based on the schematic drawing in Figure 2, consisting of three bimorphs having a length of *L*, each with an end mass and two linear springs, *K*_1_ and *K*_2_, connecting the three masses, the equations of motion for the three attached masses on the right end of the beams can be written in the following form (using the Newton’s 2nd law):

(1a) $${\overset{⌣}{V}}_{1}\left(L,t\right)-K_{1}\left[w_{1}\left(L,t\right)-w_{2}\left(L,t\right)\right]=M_{1}\cdot {\ddot{w}}_{1}\left(L,t\right)\;$$

(1b) $${\overset{⌣}{V}}_{2}\left(L,t\right)+K_{1}\left[w_{1}\left(L,t\right)-w_{2}\left(L,t\right)\right]-K_{2}\left[w_{2}\left(L,t\right)-w_{3}\left(L,t\right)\right]=M_{2}\times {\ddot{w}}_{2}\left(L,t\right)\;$$

(1c) $${\overset{⌣}{V}}_{3}\left(L,t\right)+K_{2}\left[w_{2}\left(L,t\right)-w_{3}\left(L,t\right)\right]=M_{3}\times {\ddot{w}}_{3}\left(L,t\right)\;$$

Note that ${\overset{⌣}{V}}_{1}$, ${\overset{⌣}{V}}_{2}$, and ${\overset{⌣}{V}}_{3}$ represent the shear force at each free end of the three bimorphs, respectively.

Assuming that the variables in Equations (1a)–(1c) can be written in a complex notation as (see also Appendix A).

(2) {wi(L,t),V⌣i(L,t)}=Re{Wi(L),V⌢i(L)}eiωt,i=1,2,3 

The following three equations of motion after casting the shear boundary conditions at *x* = *L*, for each of the three bimorphs, can be written as

(3a) $${\overset{⌢}{V}}_{1}\left(L\right)=K_{1}\left[W_{1}\left(L\right)-W_{2}\left(L\right)\right]-\omega^{2}\times M_{1}\times W_{1}\left(L\right)\;$$

(3b) $${\overset{⌢}{V}}_{2}\left(L\right)=-K_{1}\left[W_{1}\left(L\right)-W_{2}\left(L\right)\right]+K_{2}\left[W_{2}\left(L\right)-W_{3}\left(L\right)\right]-\omega^{2}\times M_{2}\times W_{2}\left(L\right)\;$$

(3c) $${\overset{⌢}{V}}_{3}\left(L\right)=-K_{2}\left[W_{2}\left(L,t\right)-W_{3}\left(L,t\right)\right]-\omega^{2}\times M_{3}\times W_{3}\left(L\right)\;$$

According to Appendix A, the differential equation of motion for each bimorph is written as:

(4) $$D_{i}\frac{d^{4}W_{i}\left(x\right)}{dx^{4}}-m\omega^{2}W_{i}\left(x\right)=0,\;i=1,2,3\;$$

where (see also Equations (7A) and (10A) in Appendix A)

(5) $$D_{i}={\left\{\frac{2}{3}\times E_{substrate}\times {\left(\frac{H}{2}\right)}^{3}+\frac{2}{3}\times E_{piezo}\left[{\left(\frac{H}{2}+h\right)}^{3}-{\left(\frac{H}{2}\right)}^{3}\right]\right\}}_{i}\times b_{i},\;i=1,2,3$$

with H and h, representing the substrate thickness and the piezo layer thickness, respectively and the total mass of the bimorph is given by

(6) $$m_{i}={\left(H\times b\times \rho_{substrate}+2\times h\times b\times \rho_{piezo}\right)}_{i},\begin{array}{cc} & i=1,2,3 \end{array}$$

**Figure A1:**
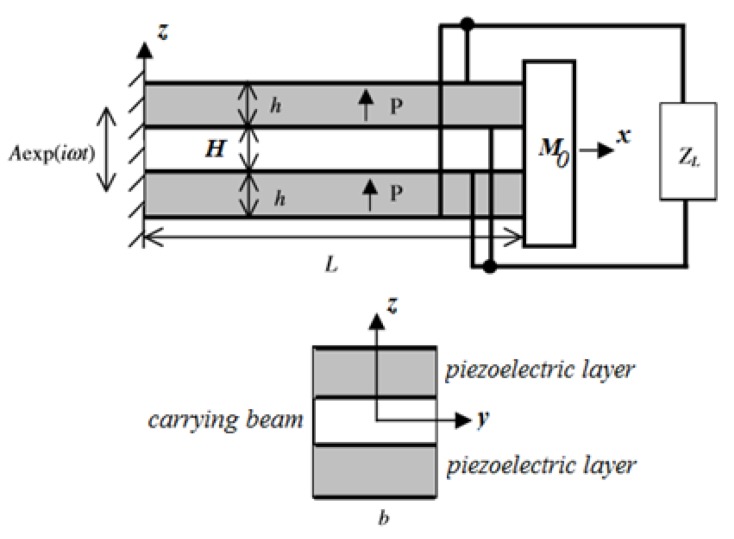
The piezoelectric bimorph model.

$\rho_{substrate}$ and $\rho_{piezo}$ being the substrate layer density and piezo layer density, respectively, and b is the width of the bimorph. Figure A1 in Appendix A presents the various geometric dimensions of the bimorph.

The general solution for each of the three bimorph beams (Equation (4)) can be written as

(7) $$W_{i}\left(x\right)=A_{i1}\cosh(\beta_{i}x)+A_{i}{}_{2}\sinh(\beta_{i}x)+A_{i3}\cos(\beta_{i}x)+A_{i}{}_{4}\sin(\beta_{i}x),\begin{array}{cc} & i=1,2,3 \end{array}\;$$

where

(8) $$\beta_{i}^{4}\equiv {\left(\frac{m\omega^{2}}{D}\right)}_{i},\begin{array}{cc} & i=1,2,3 \end{array}\;$$

The associated boundary conditions for the three equations presented by Equation (7) are next displayed.

(9a) $$@x=0\begin{array}{cc} & W_{1}\left(0\right)=A \end{array}\begin{array}{cc} ; & W_{1}{,}_{x}\left(0\right)=0. \end{array}\;$$

(9b) $$\begin{array}{cc} & W_{2}\left(0\right)=A \end{array}\begin{array}{cc} ; & W_{2}{,}_{x}\left(0\right)=0. \end{array}\;$$

(9c) $$\begin{array}{cc} & W_{3}\left(0\right)=A \end{array}\begin{array}{cc} ; & W_{3}{,}_{x}\left(0\right)=0. \end{array}\;$$

(10a) $$\begin{array}{l} @x=L\begin{array}{cc} & -D_{1}\times W_{1}{,}_{xx}(L)+{\left[E_{piezo}d_{31}\frac{\tilde{V}}{h}\times \left(H+h\right)\times h\times b\right]}_{1}=0 \end{array};\\ \begin{array}{cccc} & & & \begin{array}{cc} & ;D_{1}\times W_{1}{,}_{xxx}(L)=K_{1}\left[W_{1}\left(L\right)-W_{2}\left(L\right)\right]-\omega^{2}\times M_{1}\times W_{1}\left(L\right). \end{array} \end{array} \end{array}$$

(10b) $$\begin{array}{l} \begin{array}{ccc} & \begin{array}{cc} \begin{array}{cc} -D_{2}\times W_{2}{,}_{xx}(L)+{\left[E_{piezo}d_{31}\frac{\tilde{V}}{h}\times \left(H+h\right)\times h\times b\right]}_{2}=0; & \end{array} & \end{array} & \end{array}\\ D_{2}\times W_{2}{,}_{xxx}(L)=-K_{1}\left[W_{1}\left(L\right)-W_{2}\left(L\right)\right]+K_{2}\left[W_{2}\left(L\right)-W_{3}\left(L\right)\right]-\omega^{2}\times M_{2}\times W_{2}\left(L\right). \end{array}$$

(10c) $$\begin{array}{l} \begin{array}{cc} & -D_{3}\times W_{3}{,}_{xx}(L)+{\left[E_{piezo}d_{31}\frac{\tilde{V}}{h}\times \left(H+h\right)\times h\times b\right]}_{3}=0 \end{array};\\ \begin{array}{cccc} & & & \begin{array}{cc} & D_{3}\times W_{1}{,}_{xxx}(L)=-K_{2}\left[W_{2}\left(L,t\right)-W_{3}\left(L,t\right)\right]-\omega^{2}\times M_{3}\times W_{3}\left(L\right). \end{array} \end{array} \end{array}\;$$

where $\tilde{V}$ represents the bimorph’s generated voltage. The voltage induced on each bimorph, due to the wall excitation is written as (see Appendix A, Equation (A28))

(11) $${\tilde{V}}_{i}=\Gamma_{i}\times \beta_{i}\times \left[-A_{i1}\sinh\left(\beta_{i}L\right)-A_{i2}\cosh\left(\beta_{i}L\right)+A_{i3}\sin\left(\beta_{i}L\right)-A_{i4}\cos\left(\beta_{i}L\right)\right],\begin{array}{cc} & i=1,2,3 \end{array}\;$$

where

(12) $$\Gamma_{i}=\frac{{\left[E_{piezo}\times d_{31}\left(\frac{H}{2}+h\right)\right]}_{i}}{{\left(\frac{1}{2z_{L}}+\frac{1}{z_{0}}\right)}_{i}},\begin{array}{cc} & i=1,2,3 \end{array}\;$$

and $z_{L}$ and $z_{0}$ are the external impedance and internal impedance of the bimorphs system, respectively. Applying the boundary conditions, we get a set of regular equations with 12 unknowns, written in a matrix form as

(13) $$[\begin{array}{cccccccccccc} 1 & 0 & 1 & 0 & 0 & 0 & 0 & 0 & 0 & 0 & 0 & 0\\ 0 & \beta_{1} & 0 & \beta_{1} & 0 & 0 & 0 & 0 & 0 & 0 & 0 & 0\\ a_{31} & a_{32} & a_{33} & a_{34} & 0 & 0 & 0 & 0 & a & 0 & 0 & 0\\ a_{41} & a_{42} & a_{43} & a_{44} & a_{45} & a_{46} & a_{47} & a_{48} & 0 & 0 & 0 & 0\\ 0 & 0 & 0 & 0 & 1 & 0 & 1 & 0 & 0 & 0 & 0 & 0\\ 0 & 0 & 0 & 0 & 0 & \beta_{2} & 0 & \beta_{2} & 0 & 0 & 0 & 0\\ 0 & 0 & 0 & 0 & a_{75} & a_{76} & a_{77} & a_{78} & 0 & 0 & 0 & 0\\ a_{81} & a_{82} & a_{83} & a_{84} & a_{85} & a_{86} & a_{87} & a_{88} & a_{89} & a_{810} & a_{811} & a_{812}\\ 0 & 0 & 0 & 0 & 0 & 0 & 0 & 0 & 1 & 0 & 1 & 0\\ 0 & 0 & 0 & 0 & 0 & 0 & 0 & 0 & 0 & \beta_{3} & 0 & \beta_{3}\\ 0 & 0 & 0 & 0 & 0 & 0 & 0 & 0 & a_{119} & a_{1110} & a_{1111} & a_{1112}\\ 0 & 0 & 0 & 0 & a_{125} & a_{126} & a_{127} & a_{128} & a_{129} & a_{1210} & a_{1211} & a_{1212} \end{array}]\left\{\begin{array}{c} A_{11}\\ A_{12}\\ A_{13}\\ A_{14}\\ A_{21}\\ A_{22}\\ A_{23}\\ A_{24}\\ A_{31}\\ A_{32}\\ A_{33}\\ A_{34} \end{array}\right\}=\left\{\begin{array}{c} A\\ 0\\ 0\\ 0\\ A\\ 0\\ 0\\ 0\\ A\\ 0\\ 0\\ 0 \end{array}\right\}$$

The various terms appearing in the matrix are

(14a) $$\begin{array}{l} a_{31}=-D_{1}\times \beta_{1}^{2}\cdot \cosh\left(\beta_{1}L\right)-\chi_{1}\times \sinh\left(\beta_{1}L\right);\\ a_{32}=-D_{1}\times \beta_{1}^{2}\cdot \sinh\left(\beta_{1}L\right)-\chi_{1}\times \cosh\left(\beta_{1}L\right);\\ a_{33}=+D_{1}\times \beta_{1}^{2}\cdot \cos\left(\beta_{1}L\right)+\chi_{1}\times \sin\left(\beta_{1}L\right);\\ a_{34}=+D_{1}\times \beta_{1}^{2}\cdot \sin\left(\beta_{1}L\right)-\chi_{1}\times \cos\left(\beta_{1}L\right). \end{array}\;$$

where

(14b) $$\chi_{1}={\left[E_{piezo}\times d_{31}\times \left(H+h\right)\times b\right]}_{1}\times \Gamma_{1}\;$$

(14c) $$\begin{array}{l} a_{41}=+\alpha_{1};\begin{array}{cc} & a_{42}=+\alpha_{2};\begin{array}{cc} & a_{43}=+\alpha_{3}; \end{array}\begin{array}{cc} & a_{44}=+\alpha_{4};\begin{array}{cc} & a_{45}=+K_{1}\times \cosh\left(\beta_{2}L\right); \end{array} \end{array} \end{array}\\ a_{46}=+K_{1}\times \sinh\left(\beta_{2}L\right);\begin{array}{cc} & a_{47}=+K_{1}\times \cos\left(\beta_{2}L\right); \end{array}\begin{array}{cc} & a_{48}=+K_{1}\times \sin\left(\beta_{2}L\right). \end{array} \end{array}\;$$

with

(14d) $$\begin{array}{l} \alpha_{1}=D_{1}\times \beta_{1}^{3}\times \sinh\left(\beta_{1}L\right)-\left(K_{1}-\omega^{2}\times M_{1}\right)\times \cosh\left(\beta_{1}L\right);\\ \alpha_{2}=D_{1}\times \beta_{1}^{3}\times \cosh\left(\beta_{1}L\right)-\left(K_{1}-\omega^{2}\times M_{1}\right)\times \sinh\left(\beta_{1}L\right);\\ \alpha_{3}=D_{1}\times \beta_{1}^{3}\times \sin\left(\beta_{1}L\right)-\left(K_{1}-\omega^{2}\times M_{1}\right)\times \cos\left(\beta_{1}L\right);\\ \alpha_{4}=-D_{1}\times \beta_{1}^{3}\times \cos\left(\beta_{1}L\right)-\left(K_{1}-\omega^{2}\times M_{1}\right)\times \sin\left(\beta_{1}L\right). \end{array}\;$$

(14e) $$\begin{array}{l} a_{81}=+K_{1}\times \cosh\left(\beta_{1}L\right);\begin{array}{cc} & a_{82}=+K_{1}\times \sinh\left(\beta_{1}L\right);\begin{array}{cc} & a_{83}=+K_{1}\times \cos\left(\beta_{1}L\right); \end{array} \end{array}\\ a_{84}=+K_{1}\times \sin\left(\beta_{1}L\right);\begin{array}{cc} & a_{85}=\alpha_{5};\begin{array}{cc} & a_{86}=\alpha_{6};\begin{array}{cc} & a_{87}=\alpha_{7}; \end{array}\begin{array}{cc} & a_{88}=\alpha_{8}; \end{array} \end{array} \end{array}\\ a_{89}=-K_{2}\times \cosh\left(\beta_{3}L\right);\begin{array}{cc} & a_{810}=-K_{2}\times \sinh\left(\beta_{3}L\right);\begin{array}{cc} & a_{811}=-K_{2}\times \cos\left(\beta_{3}L\right); \end{array} \end{array}\\ a_{812}=-K_{2}\times \sin\left(\beta_{3}L\right). \end{array}\;$$

and

(14f) $$\begin{array}{l} \alpha_{5}=D_{2}\times \beta_{2}^{3}\times \sinh\left(\beta_{2}L\right)-\left[\left(K_{1}+K_{2}\right)-\omega^{2}\times M_{2}\right]\times \cosh\left(\beta_{2}L\right);\\ \alpha_{6}=D_{2}\times \beta_{2}^{3}\times \cosh\left(\beta_{2}L\right)-\left[\left(K_{1}+K_{2}\right)-\omega^{2}\times M_{2}\right]\times \sinh\left(\beta_{2}L\right);\\ \alpha_{7}=D_{2}\times \beta_{2}^{3}\times \sin\left(\beta_{2}L\right)-\left[\left(K_{1}+K_{2}\right)-\omega^{2}\times M_{2}\right]\times \cos\left(\beta_{2}L\right);\\ \alpha_{8}=-D_{2}\times \beta_{2}^{3}\times \cos\left(\beta_{2}L\right)-\left[\left(K_{1}+K_{2}\right)-\omega^{2}\times M_{2}\right]\times \sin\left(\beta_{2}L\right). \end{array}\;$$

(14g) $$\begin{array}{l} a_{75}=-D_{2}\times \beta_{2}^{2}\times \cosh\left(\beta_{2}L\right)-\chi_{2}\times \sinh\left(\beta_{2}L\right);\\ a_{76}=-D_{2}\times \beta_{2}^{2}\times \sinh\left(\beta_{2}L\right)-\chi_{2}\times \cosh\left(\beta_{2}L\right);\\ a_{77}=+D_{2}\times \beta_{2}^{2}\times \cos\left(\beta_{2}L\right)+\chi_{2}\times \sin\left(\beta_{2}L\right);\\ a_{78}=+D_{2}\times \beta_{2}^{2}\times \sin\left(\beta_{2}L\right)-\chi_{2}\times \cos\left(\beta_{2}L\right). \end{array}\;$$

where

(14h) $$\chi_{2}={\left[E_{piezo}\times d_{31}\times \left(H+h\right)\times b\right]}_{2}\times \Gamma_{2}\;$$

(14i) $$\chi_{3}={\left[E_{piezo}\times d_{31}\times \left(H+h\right)\times b\right]}_{3}\times \Gamma_{3}\;$$

and

(14j) $$\begin{array}{l} a_{119}=-D_{3}\times \beta_{3}^{2}\times \cosh\left(\beta_{3}L\right)-\chi_{3}\times \sinh\left(\beta_{3}L\right);\\ a_{1110}=-D_{3}\times \beta_{3}^{2}\times \sinh\left(\beta_{3}L\right)-\chi_{3}\times \cosh\left(\beta_{3}L\right);\\ a_{1111}=+D_{3}\times \beta_{3}^{2}\times \cos\left(\beta_{3}L\right)+\chi_{3}\times \sin\left(\beta_{3}L\right);\\ a_{1112}=+D_{3}\times \beta_{3}^{2}\times \sin\left(\beta_{3}L\right)-\chi_{3}\times \cos\left(\beta_{3}L\right). \end{array}\;$$

(14k) $$\begin{array}{l} a_{125}=+K_{2}\times \cosh\left(\beta_{2}L\right)\begin{array}{cc} & a_{126}=+K_{2}\times \sinh\left(\beta_{2}L\right);\begin{array}{cc} & a_{127}=+K_{2}\times \cos\left(\beta_{2}L\right); \end{array} \end{array}\\ a_{128}=+K_{2}\times \sin\left(\beta_{2}L\right);\begin{array}{cc} & a_{129}=+\alpha_{9};\begin{array}{cc} & a_{1210}=+\alpha_{10};\begin{array}{cc} & a_{1211}=+\alpha_{11}; \end{array}\begin{array}{cc} & a_{1212}=+\alpha_{12}. \end{array} \end{array} \end{array} \end{array}\;$$

with

(14l) $$\begin{array}{l} \alpha_{9}=D_{3}\times \beta_{3}^{3}\times \sinh\left(\beta_{3}L\right)-\left(K_{2}-\omega^{2}\times M_{3}\right)\times \cosh\left(\beta_{3}L\right);\\ \alpha_{10}=D_{3}\times \beta_{3}^{3}\times \cosh\left(\beta_{3}L\right)-\left(K_{2}-\omega^{2}\times M_{3}\right)\times \sinh\left(\beta_{3}L\right);\\ \alpha_{11}=D_{3}\times \beta_{3}^{3}\times \sin\left(\beta_{3}L\right)-\left(K_{2}+\omega^{2}\times M_{3}\right)\times \cos\left(\beta_{3}L\right);\\ \alpha_{12}=-D_{3}\times \beta_{3}^{3}\times \cos\left(\beta_{3}L\right)-\left(K_{2}+\omega^{2}\times M_{3}\right)\times \sin\left(\beta_{3}L\right). \end{array}\;$$

Once the coefficients of the three equations appearing in Equation (7), *A_i_*_1_, *A_i_*_2_, *A_i_*_3_, and *A_i_*_4_ (a total of 12 terms) are found, the voltages generated on each bimorph (Equation (11)) can be evaluated, and the harvested power can be calculated for a given excitation frequency, ω.

The power would be calculated using the following expression:

(15) $$P_{i}=I_{i}\times V_{i}\;$$

The natural frequencies of the system can be found by demanding the vanishing of the determinant of the coefficients matrix. Accordingly, a code was written within MATLAB (www.mathworks.com/products/matlab/) that calculates the natural frequencies of the system, the various coefficients, and the harvested power under a given excitation frequency. Damping is included in the analytical model by allowing the elastic compliance to have complex values. Therefore, $s_{11}$ (see Equation (A1) in Appendix A) will be replaced by $s_{11}=s_{11}\left(1-iQ^{-1}\right)$ (see a discussion in [24,41]), where *Q* is the quality factor of the bimorph (assumed to be *Q* = 100, as used in preliminary calculations). 

As the quality factor is a complex number, the power calculation was further updated to be written as

(16) $$P_{i}=\frac{1}{2}\left({\bar{I}}_{i}\times V_{i}+I_{i}\times {\bar{V}}_{i}\right)\;$$

where $\bar{I}$ and $\bar{V}$ are the conjugate numbers of $I_{i}$ and $V_{i}$, respectively.

**Figure 3:**
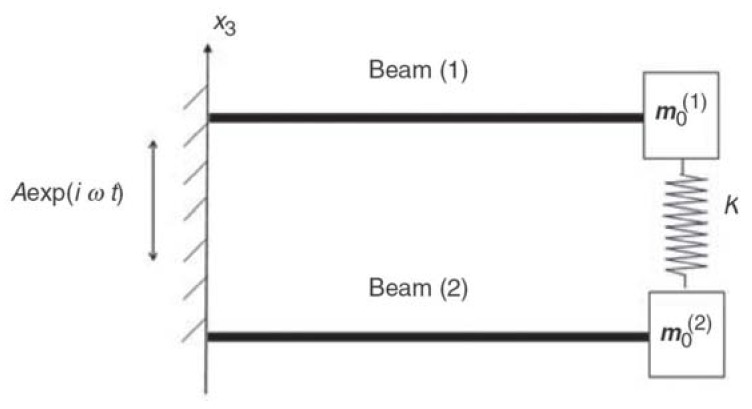
Two bimorphs spring connected analytic model.

One should note that the general model can be reduced to a simpler model, having only two bimorphs, as depicted in Figure 3. This would enable to compare the present derivation to the one presented in [24] and perform various parametric investigations.

The solution for the degenerated problem of two bimorphs has the following boundary conditions and generated voltage, respectively:

(17) $$@x=0\begin{array}{cc} & W_{1}\left(0\right)=A \end{array}\begin{array}{cc} ; & W_{1}{,}_{x}\left(0\right)=0. \end{array}\;$$

(18) $$\begin{array}{cc} & W_{2}\left(0\right)=A \end{array}\begin{array}{cc} ; & W_{2}{,}_{x}\left(0\right)=0. \end{array}\;$$

(19) $$\begin{array}{l} @x=L\begin{array}{cc} \begin{array}{cc} & -D_{1}\times W_{1}{,}_{xx}(L)+{\left[E_{piezo}d_{31}\frac{\tilde{V}}{h}\times \left(H+h\right)\times h\times b\right]}_{1}=0; \end{array} & \end{array}\\ \begin{array}{cccc} & & & \begin{array}{cc} \begin{array}{cc} & \;D_{1}\times W_{1}{,}_{xxx}(L)=K_{1}\left[W_{1}\left(L\right)-W_{2}\left(L\right)\right]-\omega^{2}\times M_{1}\times W_{1}\left(L\right). \end{array} & \end{array} \end{array} \end{array}\;$$

(20) $$\begin{array}{l} \begin{array}{cc} & -D_{1}\times W_{1}{,}_{xx}(L)+{\left[E_{piezo}d_{31}\frac{\tilde{V}}{h}\times \left(H+h\right)\times h\times b\right]}_{1}=0 \end{array};\\ \begin{array}{cc} \begin{array}{cc} & D_{1}\times W_{1}{,}_{xxx}(L)=-K_{1}\left[W_{1}\left(L\right)-W_{2}\left(L\right)\right]-\omega^{2}\times M_{1}\times W_{1}\left(L\right). \end{array} & \end{array} \end{array}\;$$

(21) $${\tilde{V}}_{i}=\Gamma_{i}\times \beta_{i}\times \left[-A_{i1}\sinh\left(\beta_{i}L\right)-A_{i2}\cosh\left(\beta_{i}L\right)+A_{i3}\sin\left(\beta_{i}L\right)-A_{i4}\cos\left(\beta_{i}L\right)\right],\begin{array}{cc} i=1,2 & \end{array}\;$$

**Figure 4:**
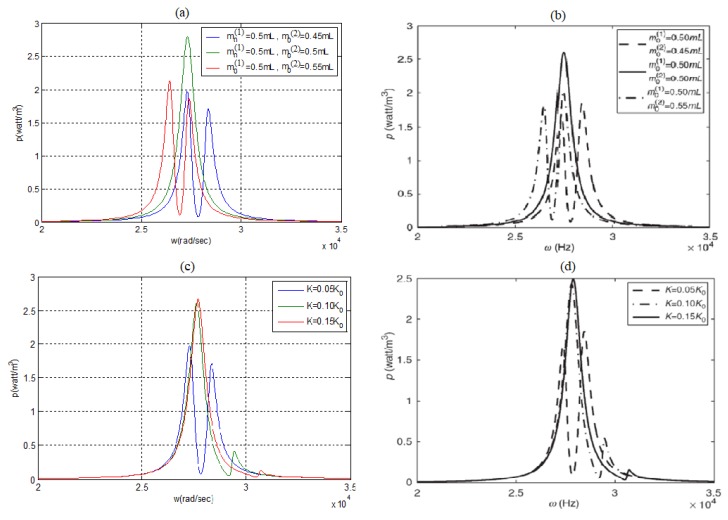
Two bimorphs interconnected by a spring-analytic power density model results for various end masses: (**a**) present model, (**b**) from [24]; for various spring constants: (**c**) present model, (**d**) from [24].

To validate the present model, a comparison was performed with the results presented in [24], yielding a good correlation between the two results, as can be observed in Figure 4a–d. The data used for comparison is presented in Appendix C and taken from [24].

One should note that, although the predictions according to the present model fit the results of [24], there are two inherent issues to be mentioned:

- In [24], there is a mistake (presumably typo) at the X axis values. The values defined as ω  Hz, are actually  ω rad/sec (see Figure 4b,d);
- The two bimorphs in [24] are electrically connected together to yield a single output voltage. As will be further described in the present manuscript, this actually reduces the output power. The correct solution, as further used in the present study, should be to individually connect each bimorph to the storage device and control the power using a smart design of the electric card.

## 3. The Experimental Campaign

This chapter is aimed at validating the derived analytical model with experiments. Three test cases were carried out: testing of the three bimorphs system, testing of the two bimorphs arrangement, and testing of the three bimorphs system with no spring connections. An experimental setup was designed and built, and various configurations of the bimorphs were tested, and their results were recorded for further processing.

### 3.1. The Experimental Setup

**Figure 5:**
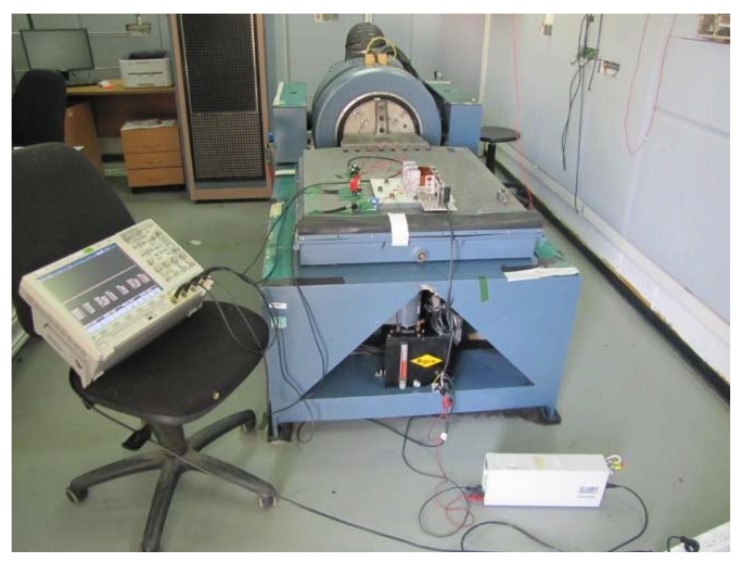
The experimental setup.

As presented in Figure 5, the experimental setup consists of the following main components:

- The piezo harvesting bimorph system;
- 1 kΩ resistance load (for each bimorph);
- A laser sensor (LG5A65PU, BANNER);
- An oscilloscope;
- A laser sensor power supply;
- A shaker table.

**Figure 6:**
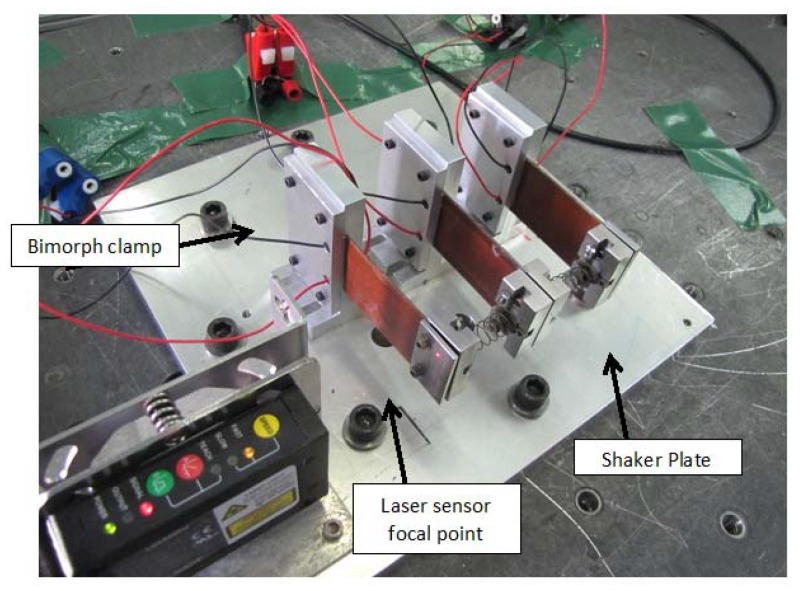
The three bimorphs system the experimental system setup.

**Figure 7:**
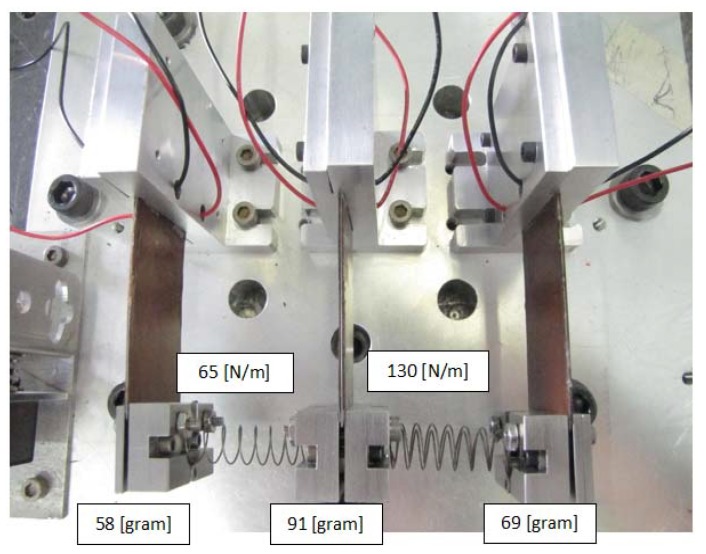
The three bimorphs system experimental setup—a zoomed view.

Each bimorph was connected separately to the oscilloscope measuring and recording its output voltage. Another channel in the oscilloscope was used to measure the laser sensor output. The laser sensor measured only the responses of two external masses (it cannot measure the response of the middle mass due to no direct line to it). Typical configurations of the three bimorphs spring connected system are presented in Figure 6 and Figure 7. Figure 6 presents the present system, while Figure 7 is zoomed on the three bimorphs, three end masses, and the two springs interconnecting them.

### 3.2. The Bimorph Beams

**Figure 8:**
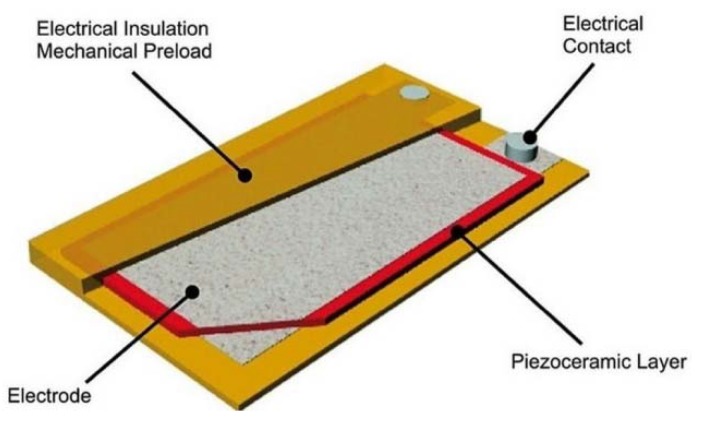
Piezo layer, P-876.A11 (from PI Ceramic website).

Each bimorph was built of two piezo layers and a 301 stainless steel strip (type 301 is an austenitic chromium-nickel stainless steel that provides high strength and good ductility when cold worked. It is a modification of Type 304 in which the chromium and nickel contents are lowered to increase the cold work-hardening range). The piezo layer was PI Ceramic P-876. A11 (www.piceramic.com/en/), which has a 0.1 mm piezo thickness (PIC255) and an electronic insulation (Kapton tape—Kapton is a polyimide film developed by DuPont in the late 1960s that remains stable across a wide range of temperatures, from −269 to +400 °C) that protects the piezo material and preloads the layer. The dimensions of the piezo patch are 61 × 35 mm^2^, while the piezo layer is only 50 × 30 mm^2^ (see Figure 8).

The end masses were manufactured from 303 stainless steel and were fixed to the bimorph with a screw and a nut to a counter plate, as can be seen in Figure 7. (Alloy 303 was specially designed to exhibit improved machinability while maintaining good mechanical and corrosion resistant properties Due to the presence of sulfur in the steel composition, Alloy 303 is the most readily machinable austenitic stainless steel; however, the sulfur addition does lower Alloy 303’sproperties.) The end mass + counter plate + screw and nut have been weighted before assembly on the bimorphs yielding a total mass of 58 gr and 69 gr for the two external bimorphs and 91 gr for the middle bimorph. The springs were manufactured from 302 stainless steel. (Alloy 302 is a variation of the 18% chromium/8% nickel austenitic alloy, which is the most familiar and the most frequently used in the stainless-steel family. Alloy 302 is a slightly higher carbon version of 304, often found in strip and wire forms.) Fixing the spring to the end mass was done by adding a small rectangle sheet that can enter between the first and final coils of the spring. The sheet was attached to the end mass by two screws.

### 3.3. Calibration of the Analytical Model Using the Experimental Test Results

Inherent differences between the analytical model and the tested one led to different performance results. Every bimorph (even designed to be similar) has its own stiffness due to gluing variance of the piezo layer to the substrate (position and amount of glue), a variation in the dimensions of the substrate due to inaccurate cutting, and disparity between the piezo layers.

The assembly of the whole system also causes some variance between the models due to the way the bimorphs are clamped and due to different assemblies of the end masses and the springs.

To be able to use the analytical model in a reliable way, the model was tuned and eventually adjusted using the experimental results by multiplying the assumed stiffness of each bimorph by a stiffness factor (experimentally found). Additionally, the Q factor (Quality factor) was also adjusted to give a range of 10–20. One should note that the Q factor mainly influences the height and width of the frequency response graph.

### 3.4. Three Bimorphs System without Spring Connections—Test Results

The aim of these tests was to calibrate the following system parameters:

- The quality factor, Q;
- The bimorph stiffness;
- The measurement of the natural frequencies for each bimorph separately.

**Figure 9:**
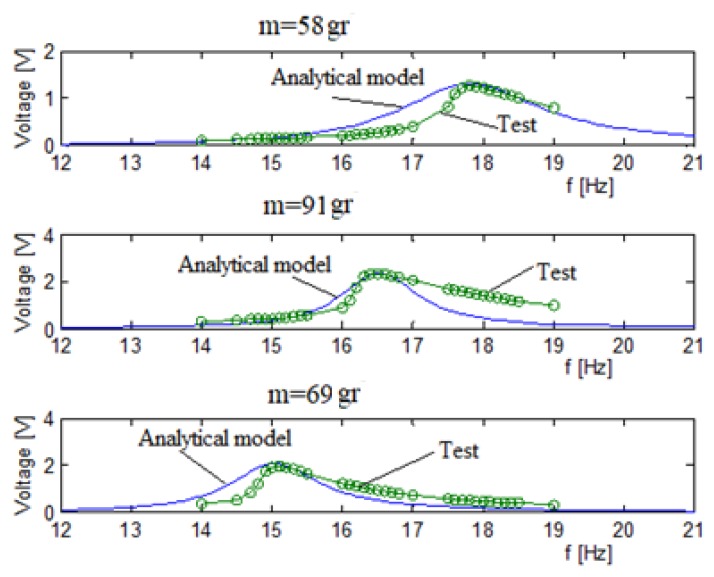
Three bimorphs without spring connection output voltage-tests validation.

Figure 9 presents the voltage versus input frequency for each bimorph separately (58 gr, 91 gr, 69 gr). The blue line is the analytic model predictions, and the green dotted line is the experimental results.

The analytic curve presented in Figure 9 is after the calibration of the Q and stiffness factors. The stiffness and Q factors, as obtained from the calibration process, are presented in Table 1. The use of these factors yields a better prediction of the analytical model, as shown in Figure 9 and Table 2.

As presented in Table 2, a very good match between the analytic model (after its tuning process) and the experimental results was obtained. The difference between the analytic model and the test result is in the range of 4% (maximal), which is small enough for engineering purposes.

A small nonlinearity can be observed in Figure 9 for the test results. It is known that piezo materials have nonlinear stress-strain curves, including a hysteresis. Therefore, a plausible explanation for the small nonlinearity encountered during the experimental campaign can be attributed to the change in the stiffness of the piezo layers for large displacements in the vicinity of the natural frequency. One should remember that one of the assumptions for the development of the analytical model, within the present study, was a linear piezo, and therefore the analytical model cannot predict the small nonlinear behavior experimentally detected.

### 3.5. Three Bimorphs Electrically Connected in Parallel with Interconnecting Springs—Experimental Results

**Figure 10:**
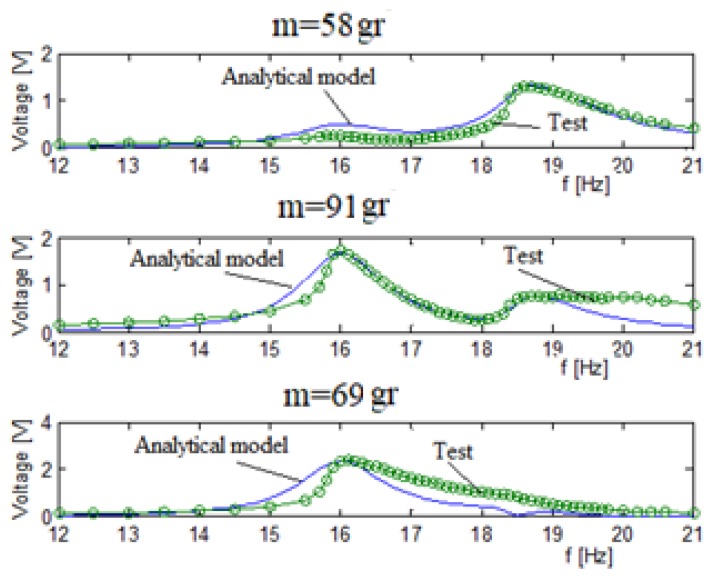
Three bimorphs system output voltage (parallel electrical connection)—experimental results vs. analytical predictions.

**Figure 11:**
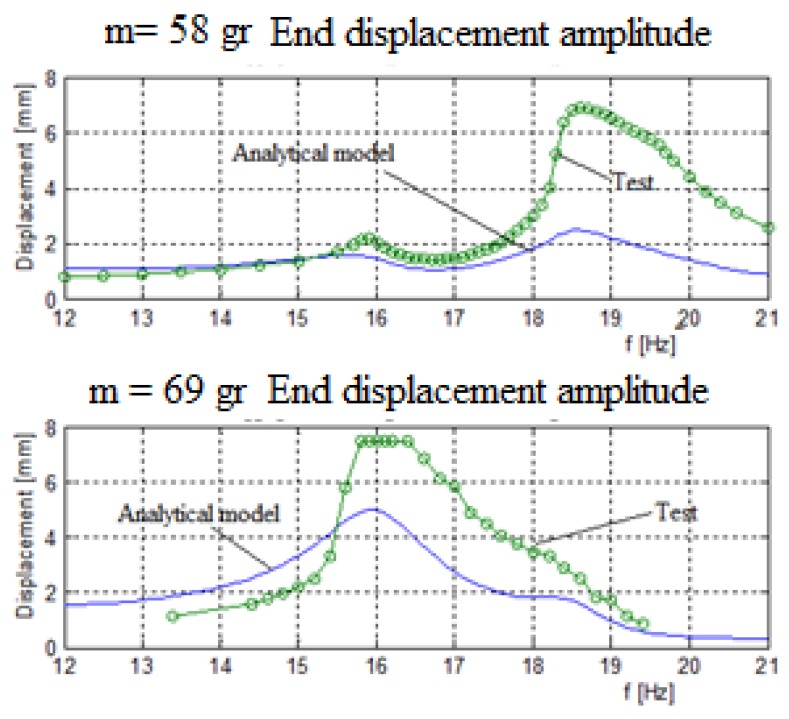
Three bimorphs system displacements (parallel electrical connection)—experimental results vs. analytical predictions.

Figure 10 displays the output voltage versus the input excitation frequency for each bimorph, separately where the blue continuous line is the analytic model and the green dotted line is the experimental results, while Figure 11 presents the comparison of the displacement amplitude for the two external end masses. The system had three end masses and two springs. The end masses were 58 gr and 69 gr for the two external bimorphs and 91 gr for the middle bimorph. The spring constant connecting the external mass (58 gr) to the middle mass (91 gr) is 65 N/m. The spring constant connecting the external mass (69 gr) to the middle mass is 130 N/m.

Note that the electrical connection for every bimorph (connecting the two piezoelectric layers sandwiching the substrate beam) was in parallel, as can be further read in Appendix B.

In general, a good matching between the predictions of the analytical and the experimental results was found, as depicted in Figure 10 and Figure 11. Note that while the matching between the analytical and experimental generated volts is very good (see Figure 11), the fitting of the relevant end displacements amplitudes is less good, with the experimental values being higher than the predicted one (probably due to high value of the damping assumed in the analytical model), mainly at the higher frequencies, although the curves’ shapes are similar. Also, one should note that the measurements of the displacement amplitude are available only for the two external masses, as the middle mass was too far from the focal point of the laser sensor and hidden by the other external masses.

The stiffness and Q factors for the three bimorphs system interconnected by two springs, as obtained from the tuning process, are shown in Table 3. Comparing the results with those presented in Table 1 reveals same values for the quality factor, Q, while the stiffness factor increased up to a value of 1.56. Remembering that the results in Table 3 are for bimorphs interconnected by springs, the increase in the stiffness factor is plausible.

Table 4 presents the voltage comparison between the predictions of the analytic model and the test results at the first and second natural frequencies. Browsing the peak voltages in Table 4 shows that the best fit for each bimorph is when the peak natural frequency is the natural frequency the bimorph is “responsible” for. Taking, for example, the first bimorph with an end mass of 58 gr, we see that the best correlation is for the second peak at the 18.8 Hz which is close to its own natural frequency (17.8 Hz). At the first peak (16 Hz), the correlation is not so good; however, it appears at minimal voltage amplitude, and therefore the influence of the miss-correlation has a small influence on the total voltage correlation.

### 3.6. Three Bimorphs Electrically Connected in Series with Interconnecting Springs—Experimental Results

**Figure 12:**
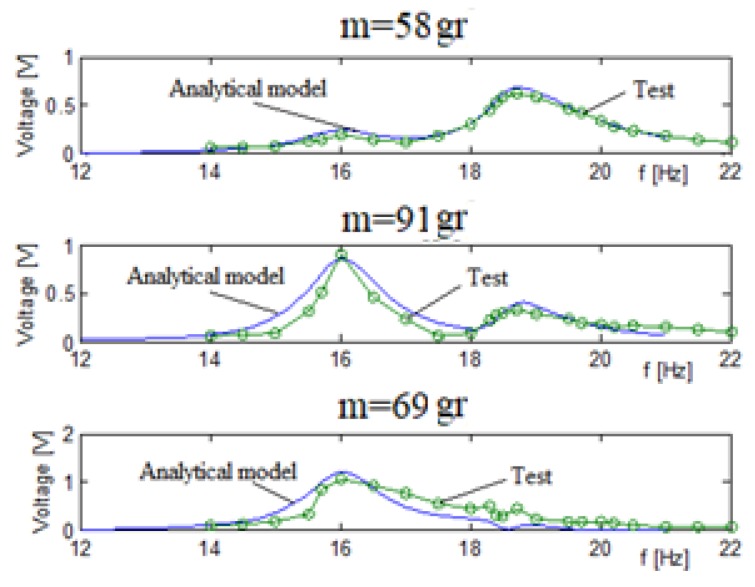
Three bimorphs system output voltage (series electrical connection)—experimental results vs analytical predictions.

To demonstrate the importance of the electrical connection (parallel vs. series) of the two piezoelectric patches forming the bimorph, a new set of tests was performed, with the piezoelectric patches being connected in series. The generated output voltage results vs. the excitation input frequency are presented in Figure 12.

Table 5 shows a relatively good match between the predictions of the analytical model and the experimental results. The generated voltage for each bimorph, and thus the total voltage of the system when each bimorph has a series connection, is smaller than the parallel connection case, up to about 1 V for the series case compared to approximately 2.3 V for the parallel connection. The natural frequencies remain the same.

The conclusion that the higher output voltage is obtained when using a parallel electric connection was the main reason for choosing it throughout all the calculations performed during the parametric study to be next presented.

### 3.7. Two Bimorphs System—Experimental Results

**Figure 13:**
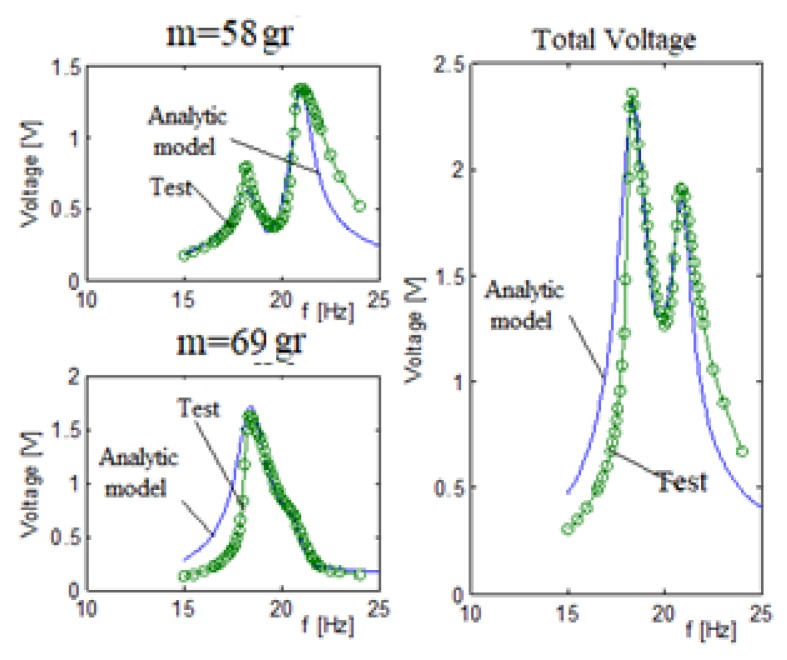
Two bimorphs system voltage—analytical vs. experimental results.

**Figure 14:**
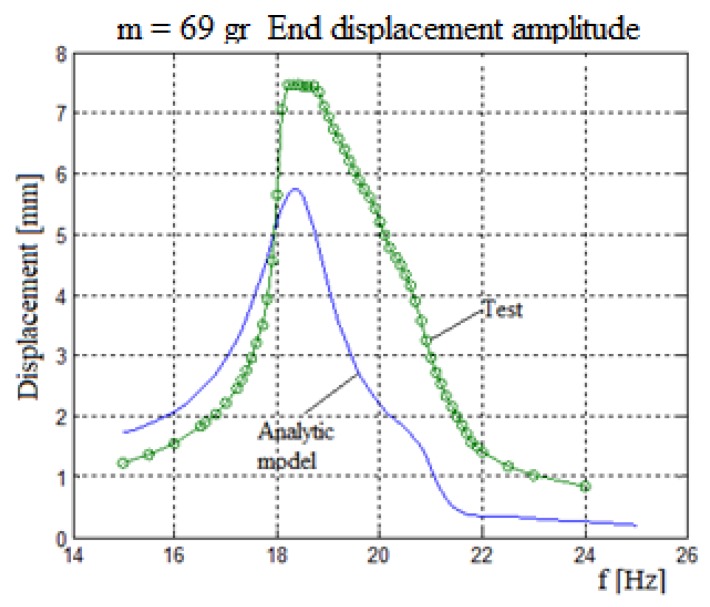
Two bimorphs system amplitude—analytical vs. experimental results.

Figure 13 presents the generated output voltage vs. the excitation input frequency for the two bimorphs system. The two bimorphs system had 58 gr and 69 gr end masses with a 65 N/m spring interconnecting it. Figure 14 displays the end mass displacement amplitude for the bimorph with a 69 gr mass.

A close look at Figure 13 reveals a good match between the analytic model predictions and the test results. Similar to the three bimorphs system, the best fit between the model and the experiment for each bimorph is at the frequencies the bimorph is “responsible” for. For this case, the best fit is for the bimorph with a 58 gr is at its second peak, while for the second bimorph with a mass of 69 gr, its best fit is at its first peak (see Table 6 and Table 7). As for the case of three bimorphs, the test results are higher than the predicted ones (probably due to a high damping ration used in the analytical model), while the shape of both curves remaining similar (see Figure 14).

### 3.8. Three vs. Two Bimorphs System

**Figure 15:**
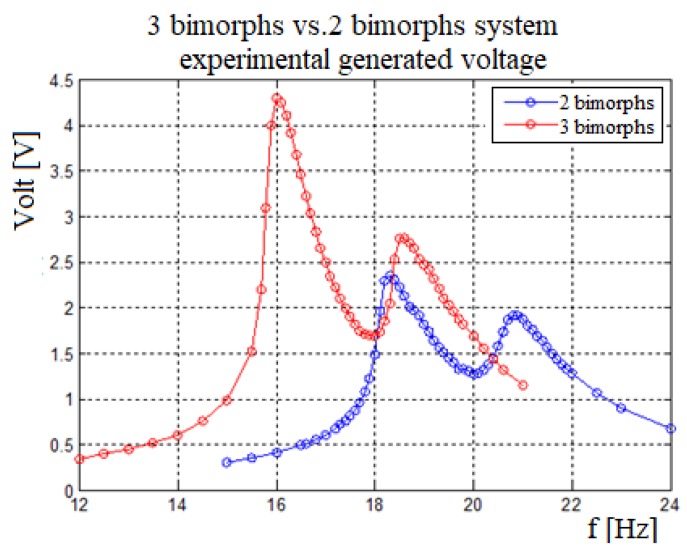
Three bimorphs vs. two bimorphs system—experimental generated voltage.

Figure 15 shows the total voltage for a three vs. two bimorphs as obtained during the experimental campaign. One can observe that the three bimorphs system has lower natural frequencies as compared to the two bimorphs system, due to its high mass middle mass and the stiffness factor that is larger than in the two bimorphs system. As expected the voltage and the bandwidth for the three bimorphs system are larger than for the two bimorphs system.

## 4. Parametric Investigation

### 4.1. Two Bimorphs System

The goal of the parametric investigation is to understand the influence of every parameter on the system’s performance and to enable the designer to choose the correct values for the various parameters of the system according to its design constraints.

The following parameters were varied:

- The spring constant;
- The weight of the end mass (see Appendix D);
- The bimorph geometry: length, width, and piezo thickness (see Appendix D).

The maximum power output is when the electric load impedance equals the source impedance. 

To understand the maximum power output that can be achieved, the parametric research will assume impedance matching, namely $Z_{L}=iZ_{0}$, where $Z_{0}=\frac{1}{i\omega C_{0}}$ and $C_{0}=\frac{\bar{\varepsilon_{33}}bL}{h}$.

The following data was used throughout the parametric investigation: length *L* = 50 mm, width *b* = 30 mm, substrate thickness *c* = H/2 = 0.1 mm, and piezoelectric layer thickness *h* = 0.1 mm. The end mass weight was 30 and/or 50 gr and the spring constant was 40 N/mm. The substrate material was chosen to be aluminum. The piezoelectric material is PZT 5 H with a quality factor of $10^{2}$.

#### The Influence of the Connecting Spring

**Figure 16:**
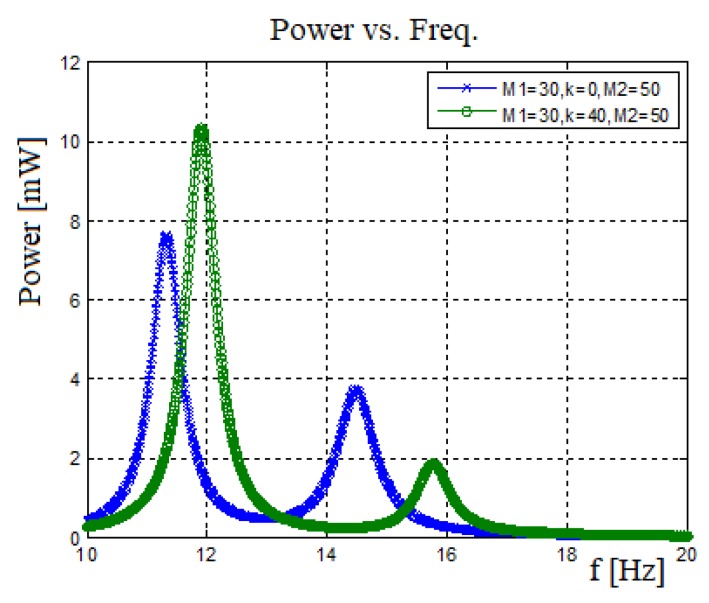
Harvested power: no spring case vs. connected spring case.

**Figure 17:**
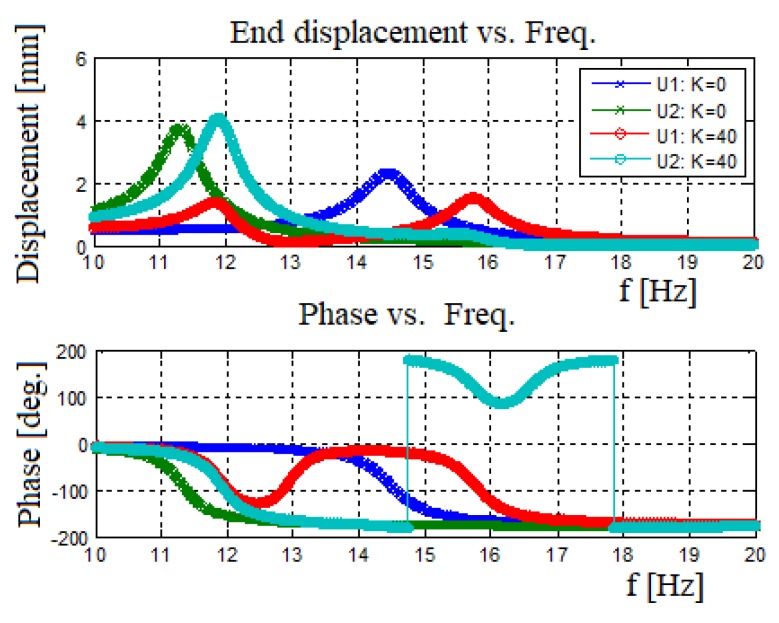
Amplitude and phase of the end mass: no spring case vs. connected spring case.

To understand the contribution of end mass spring connection, it is important to compare a system having a connecting spring with the one without the spring. Figure 16 presents the output generated power vs. input excitation frequency. Figure 17 displays the amplitude and phase versus input frequency, where U1 represents the 30 gr bimorph’s end mass amplitude. U2 represents the 50 gr bimorph’s end mass amplitude.

For the case of no spring connected between the two bimorphs, one can observe the first natural frequency for each bimorph as presented in Figure 16. In addition, Figure 16 also shows that adding a spring between the bimorphs leads to the shifting of the natural frequencies and increases the generated power. To further understand the power diagram presented in Figure 16, it is important address the graphs presented in Figure 17. This figure describes the amplitude and the phase of the end masses as a function of the input frequency. For the case without a spring, the two masses move independently. However, adding a spring between the end masses changes the whole behavior: At the system’s first natural frequency, the two masses would vibrate at the same phase (with different amplitudes) leading to higher generated voltage and power as compared to the case without a spring. At the second natural frequency, as expected, the two masses vibrate at opposite phases (with different amplitudes), leading to an effective damping (due to the opposite movements of the two masses), which reduces the displacement amplitude of the end mass in charge of that given natural frequency (in our case the beam with the smaller end mass) yielding a lower output voltage and power at the second natural frequency of the system, as compared with the case without a spring.

One should note that, for the case with a spring connecting the two bimorphs, the frequency bandwidth is expanding, causing a low power region (“power pit”) between the two natural frequencies. This region of low power gets wider as the end mass ratio gets higher and the natural frequencies get far from each other. In addition, using the phase diagram (Figure 17), one can observe that for the without the spring case, there is no connection between the two bimorph beams, and at each natural frequency, the phase is changed by 1800, keeping this phase delay throughout the whole spectrum of the exciting frequencies. The behavior changes when a spring is connected between the two end masses. The heavy mass (which causes the lower natural frequency) changes its phase by 1800 at the first system natural frequency, maintaining it till the second system natural frequency, while the second mass also changes phase, returning to 0^0^ phase of the first natural frequency. At the second system natural frequency, the lighter mass (causing this frequency) changes it phase by 1800, while the second beam slightly changes its phase, after which it is returning to its starting phase (1800 at the second natural frequency).

### 4.2. Three Bimorphs System

After completing the parametric investigation and understanding the basics of the two bimorphs system, the next step is to understand the influence of adding another bimorph at the middle on the system. The three bimorphs system investigation investigated the influence of the following parameters:

- The middle bimorph end mass;
- The two springs interconnecting the three bimorphs (see Appendix E);
- The electrical connection—every bimorph is either separately connected, or all the bimorphs are connected.

#### 4.2.1. Middle Mass Influence

For the three bimorphs system, *M*_1_ represents the smaller mass, *M*_2_ represents the middle mass, and *M*_3_ represents the external higher mass (higher than the *M*_1_ external mass). *K*_1_ represents the spring constant connecting *M*_1_ and *M*_2_ while *K*_2_ represents the spring constant connecting *M*_3_ and *M*_2_. The constant end masses are *M*_1_ = 30 gr and *M*_3_ = 50 gr. The constant springs constants are *K*_1_ = 40 N/m and *K*_2_ = 80 N/m, while the middle mas *M*_2_ will be varied. The bimorphs are identical with the previous investigated cases with *L* = 50 mm, *b* = 30 mm, *c* = 0.1 mm, and *h* = 0.1 mm. The influence of the middle mass on the performance of the present harvester is summarized in Table 8.

**Figure 18:**
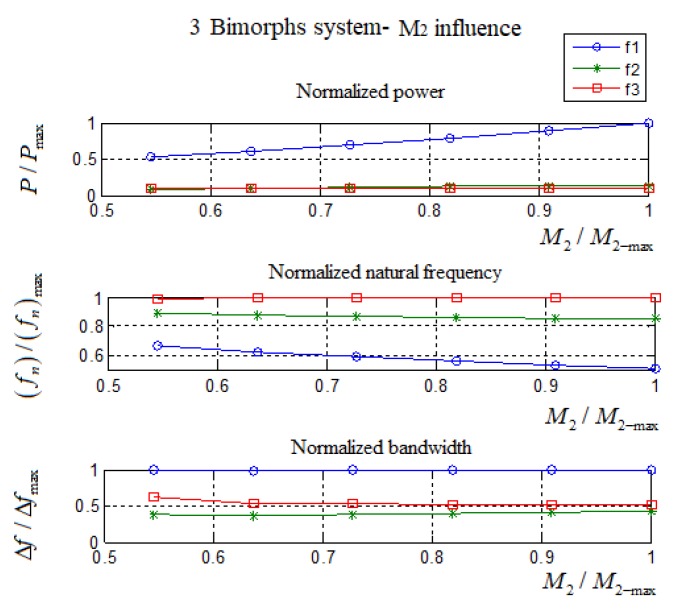
The influence of the normalized middle mass, *M*_2_, on the output harvested power, natural frequency, and bandwidth (all normalized)—three bimorphs interconnected by two springs case.

One should note that, for this set of parameters, the second and third peaks were too close to each other, so between the two peaks, the minimum power output was above 1 mW. Thus, the point where the minimum power output value between the two peaks had been chosen to be one side of the bandwidth and the other point was the original 1 mW. For design purposes the values in Table 8 were normalized and presented in Table 9 and Figure 18, where the values on the X axis were normalized by the maximum mass weight $M_{2}{}_{max}=$110 gr. (Note that due to the explanations (*) and (**), in Table 8, the normalization included only *M*_2_ = 60 gr–110 gr values.) 

From Table 8 and Table 9 and Figure 18, one can observe that a raise of the middle mass, M_2,_ will have the following effects:

- The first and second natural frequencies decrease while the third one increases slightly. As the middle mass becomes heavier, its self-natural frequency decreases, and thus, the system natural frequency decreases. On the other hand, the slightly increase in the third natural frequency is achieved due to the growing distance between the self-natural frequency of each bimorph (without a spring connection). Thus, the influence of the higher mass bimorph (middle mass) at the lower mass bimorph is decreasing as the mass value increases.
- The generated power at the first natural frequency increases, while the power at the second and third remain the same due to the same explanation presented above for the natural frequencies.
- The bandwidth at the first natural frequency is almost constant, while at the second natural frequency, it increases, and at the third, it moderately decreases.

It is interested to note that, due to the decrease of the first natural frequency and the increase of the third natural frequency, the system shows a total increase in the system’s bandwidth. This comes with the disadvantage of “power pit” regions between the natural frequencies.

#### 4.2.2. Three Bimorphs System vs. Two Bimorphs System

**Figure 19:**
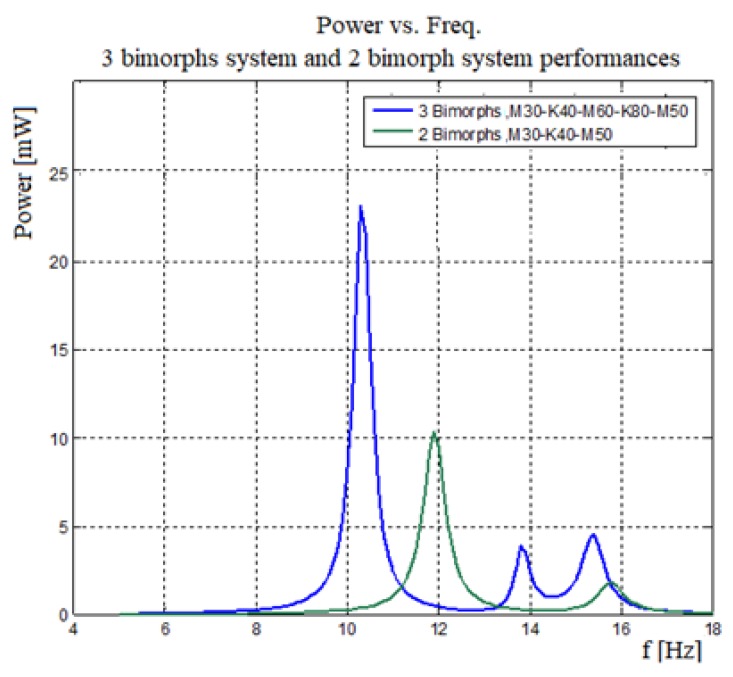
Generated power vs. frequency—three bimorphs and two bimorphs performances.

The main objective of the present study was to expand the power output bandwidth. A system having more bimorphs is expected to have a wider bandwidth. Figure 19 presents a performance comparison between three bimorphs system and two bimorphs system.

As depicted in Figure 19, the three bimorphs system has a wider bandwidth compared to that of two bimorphs system. Moreover, the three bimorphs system has a larger output power, and by adding an additional bimorph (the middle one), the width of the “power pit” is reduced. Similar results were published in [26,37].

#### 4.2.3. Bimorphs’ Electrical Individual vs. Together

The bimorphs forming the harvester can be electrically connected together and then to the electrical circuit, or each bimorph can be connected individually to the electrical circuit and the output from all the bimorphs directed to the storing system. The connected systems assume that all the plus signs are connected to each other and all the minus signs are connected to each other. The individual systems assume that every bimorph is electrically on its own, and the central system electric circuit is the responsible for the sum of the voltage and current and power output.

**Figure 20:**
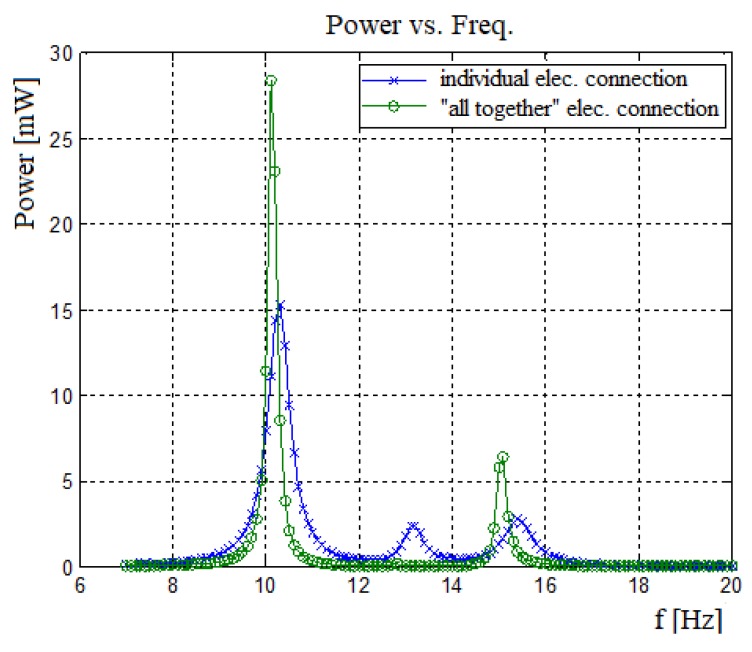
Power output for electrically individual connection vs. “all together” connected bimorphs—a three bimorphs system (*L* = 50 mm, *b* = 30 mm, *c* = 0.1 mm, *M*_1_ = 30 gr, *M*_2_ = 60 gr, *M*_3_ = 50 gr, *K*_1_ = 40 N/m, and *K*_2_ = 60 N/m).

As shown in Figure 20, the power output obtained by “all together” electrical connection of the three bimorphs is less than the individual type connection (except for the first and third natural frequencies). Although the output power for the first and third natural frequencies is higher for the “all together”–type electrical connections (due to larger displacement amplitudes), the bandwidth is very narrow, the “power pit” between the two frequencies is wider and the power for the second natural frequency is almost negligible. Summing up the individual type power outputs will result in more generated power as compared with the “all together” electrical connection approach.

## 5. Conclusions and Summary

The present study focuses on the development, manufacturing, and testing of an advanced harvesting system based on three bimorphs, capable of adjusting their natural frequencies using tip end masses and interconnected by springs, thus enlarging the system’s bandwidth.

An analytical model was developed for the three bimorphs interconnected by two springs with three end masses. The model can predict the output generated voltage from each bimorph and then the total output power is measured on a given outside resistor as a function of the material properties, the geometric dimensions of the vibrating beams, the end masses, and the spring constants.

The analytical model was then compared with data in the literature, yielding a good correlation.

To further increase the reliability of the model, a test set-up was designed and manufactured that included three bimorphs with three end-masses connected by two springs. The system was excited using a shaker, and the output voltage was measured for each bimorph for various configurations. Then, the analytical model was tuned based on the test results by introducing two factors—the quality and the stiffness factors—and the predictions of the calibrated analytical model were compared with the experimental results yielding a good correlation.

The calibrated analytical model was then used to perform a comprehensive parametric investigation for two and three bimorphs systems, in which the influences of various parameters—like spring constant, mass value, thickness, width, and length of the bimorph and the substrate beam, as well as the way the bimorphs are electrically connected on the output generated power—were investigated. 

It turned out that the connection spring constant is crucial. If the spring constant is too small compared to the bimorph’s stiffness than, its influence is negligible, and the system acts like there is no spring (*K* = 0). On the other hand, spring constant too stiff compared to the bimorph’s stiffness makes the spring acting like a rigid bar. Thus, for the two bimorphs system, for example, the second natural frequency would vanish. A preferred spring constant would be about 15% from the stiffness of the bimorph ($0.15\frac{3EI}{L^{3}}$).

As the mass ratio between the 2 bimorphs increases the “power pit” increases, which reduces the system effectiveness. Decreasing the mass ratio decreases the “power pit” but narrows the bandwidth. Thus, the selection of the mass ratio is critical. A preferred end mass ratio should be 10–20, depending on system geometry and spring rate. The same conclusion can be found in [26,37]. Note that a complete disappearance of the power pit can be achieved only for the case of two very close natural frequencies, yielding a narrow bandwidth, and therefore not applicable. The designer would have to choose the harvesters parameters in such a way to minimize the width of the power pit while maximizing the bandwidth.

As predicted, the three bimorphs system generates higher power and wider bandwidth compared to the two bimorphs system. Adding more bimorphs and springs to the system is expected to increase even more the generated output power as well as the bandwidth of the system.

The three bimorphs system presents larger degrees of freedom for the designer as compared with the two bimorphs system. Thus, the designer will be able to deal better with the “power pit” issue and to correctly adjust the natural frequencies to the expected excitation input.

The “all together” vs. individual electrical connection for the various bimorphs forming the harvester system was parametrically investigated showing that bimorphs’ individual electrical connections and then summing up their generated power would yield a better harvester. This conclusion is in line to a similar statement presented in [35].

## Figures and Tables

**Table 1:** Three bimorphs system without spring connections-stiffness and Q factors.

Bimorph No.	Stiffness Factor	Quality Factor
1	1.13	5.85
2	1.5	10
3	0.95	7.9

**Table 2:** Three bimorphs system without spring connections (*K*_1_ = *K*_2_ = 0)—analytical predictions vs. experimental results.

Value	Bimorph 1	Bimorph 2	Bimorph 3
	58 gr	91 gr	69 gr
	Natural Frequency-17.8 Hz	Natural Frequency-16.5 Hz	Natural Frequency-15.1 Hz
Analytic model V	1.304	2.368	2.042
Test result V	1.26	2.32	1.965
Analytic model/test result ratio	1.035	1.02	1.039

**Table 3:** Three bimorphs system-stiffness and Q factors.

Bimorph No.	Stiffness Factor	Q Factor
1	1.21	6.8
2	1.56	10
3	1.02	7.9

**Table 4:** Experimental results vs. analytical predictions (parallel electrical connection)—the three bimorphs system.

Value	Bimorph 1		Bimorph 2		Bimorph 3	
	58 gr		91 gr		69 gr	
	1st Peak 16 Hz	2nd Peak 18.8 Hz	1st Peak 16 Hz	2nd Peak 18.8 Hz	1st Peak 16 Hz	2nd Peak 18.8 Hz
Analytic model V	0.494	1.303	1.661	0.7619	2.326	0.1895
Test result V	0.25	1.28	1.71	0.765	2.335	0.605
Analytic model/test result ratio	1.977	1.017	0.971	0.9959	0.9961	0.11

**Table 5:** Experimental results vs. analytical predictions (series electrical connection)—the three bimorphs system.

Value	Bimorph 1		Bimorph 2		Bimorph 3	
	58 gr		91 gr		69 gr	
	1st Peak	2nd Peak	1st Peak	2nd Peak	1st Peak	2nd Peak
Analytic model V	0.2459	0.6812	0.856	0.3931	1.202	0.0664
Test result V	0.195	0.625	0.9	0.325	1.055	0.45
Analytic model/test result ratio	1.26	1.089	0.95	1.2	1.13	0.147

**Table 6:** Two bimorphs system—stiffness and Q factors.

Bimorph No.	Stiffness Factor	Quality Factor
1	1.55	20
2	1.5	11

**Table 7:** Two bimorph system—model predictions vs. experimental results (voltage).

Value	58 gr		69 gr	
	1st Peak 18.3 Hz	2nd Peak 20.6 Hz	1st Peak 18.3 Hz	2nd Peak 20.6 Hz
Analytic model V	0.6344	1.381	1.630	0.5633
Test result V	0.740	1.025	1.615	0.710
Analytic model/test result ratio	0.857	1.347	1.009	0.793

**Table 8:** Three bimorphs system—the influence of the middle mass, M_2_.

*M*_2_	f Hz			Power mW			Δf Hz		
	1st Peak	2nd Peak	3rd Peak	1st Peak	2nd Peak	3rd Peak	1st Peak	2nd Peak	3rd Peak
30	12.02		15.91	14.98		1.901	1.91		1.71
40	11.56		15.29	17.49		1.175	2.1		1.15 *
50	10.98	14.39	15.17	20.43	7.691	5.848	2.19	0.71 **	1.1 **
60	10.33	13.83	15.38	23.46	3.94	4.502	2.2	0.85	1.37
70	9.72	13.57	15.44	26.86	4.401	4.373	2.18	0.8	1.19
80	9.19	13.43	15.47	30.68	4.917	4.353	2.19	0.85	1.16
90	8.72	13.34	15.49	34.89	5.328	4.354	2.19	0.88	1.14
100	8.31	13.27	15.5	39.43	5.646	4.363	2.21	0.92	1.14
110	7.95	13.23	15.51	44.25	5.894	4.372	2.22	0.95	1.13

(*) This set of parameters made smaller power output. Thus, the bandwidth calculation was for 0.5 mW. (**) Normally, at this study, the chosen parameters produced between every two natural frequencies an area of frequencies that had a power output less than 1 mW. Thus, the calculation of the bandwidth for each natural frequency was simple (the area that had above than 1 mW power).

**Table 9:** Three bimorphs system—the influence of the middle mass, *M*_2_, normalized values.

$\;M_{2}/M_{2}{}_{max}\;$	$\;f_{n}/f_{n}{}_{max}\;$			$\;P/P_{max}\;$			$\;\Delta f/\Delta f_{max}\;$		
	1st Peak	2nd Peak	3rd Peak	1st Peak	2nd Peak	3rd Peak	1st Peak	2nd Peak	3rd Peak
0.545	0.666	0.892	0.992	0.530	0.089	0.102	0.991	0.383	0.617
0.636	0.627	0.875	0.995	0.607	0.099	0.099	0.982	0.360	0.536
0.727	0.593	0.866	0.997	0.693	0.111	0.098	0.986	0.383	0.523
0.818	0.562	0.860	0.999	0.788	0.120	0.098	0.986	0.396	0.514
0.909	0.536	0.856	0.999	0.891	0.128	0.099	0.995	0.414	0.514
1.000	0.513	0.853	1.000	1.000	0.133	0.099	1.000	0.428	0.509

**Figure A2:**
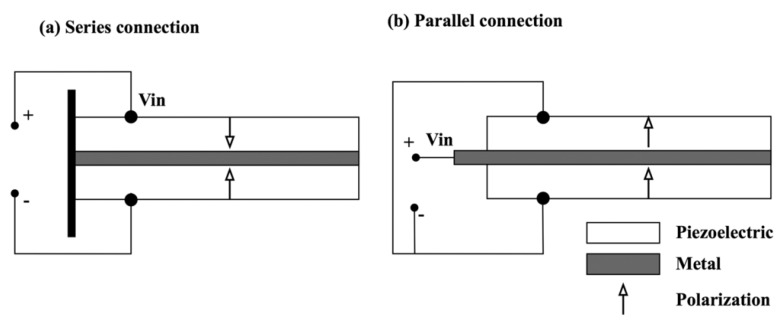
Piezoelectric bimorph connected in series and in parallel.

**Figure A3:**
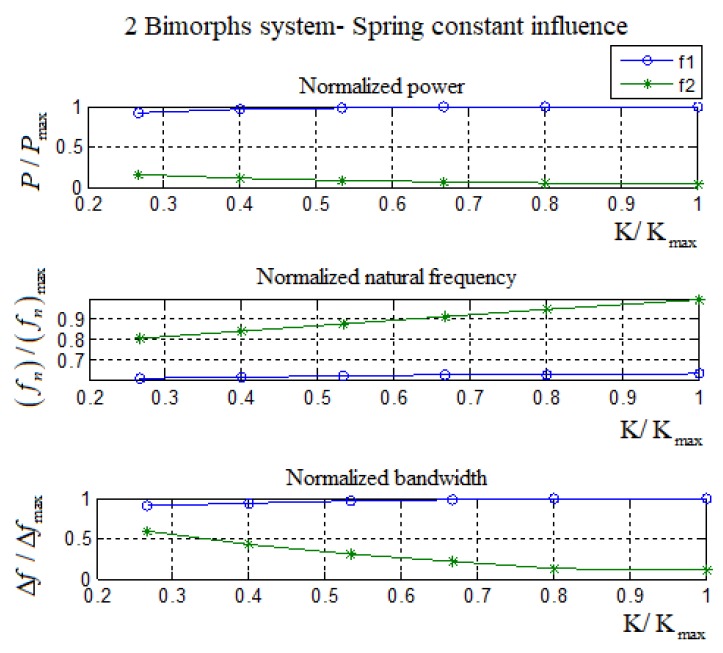
The influence of the normalized spring constant on the output power, the natural frequencies, and the bandwidth (all normalized)—two bimorphs interconnected by a spring case.

**Figure A4:**
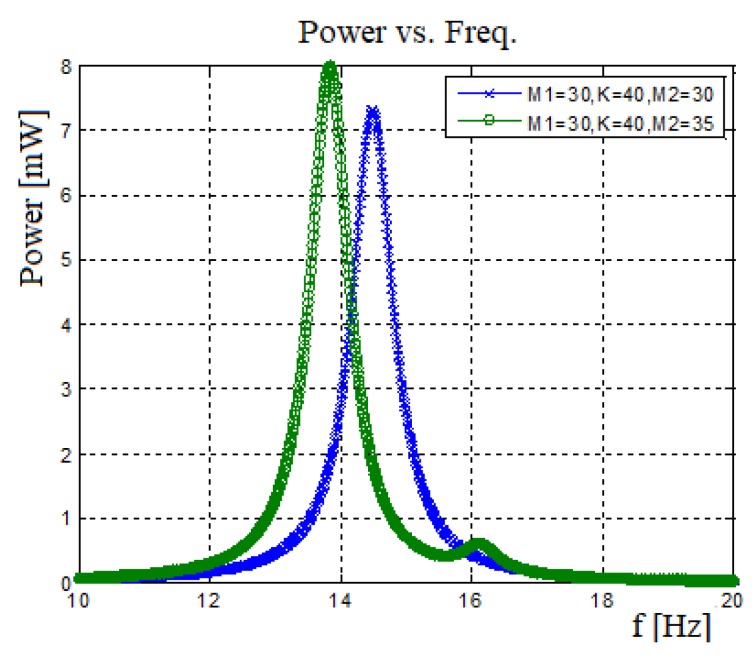
Harvested power—two bimorphs interconnected by a spring case-end mass influence: *M*_2_ = 30 gr & *M*_2_ = 35 gr.

**Figure A5:**
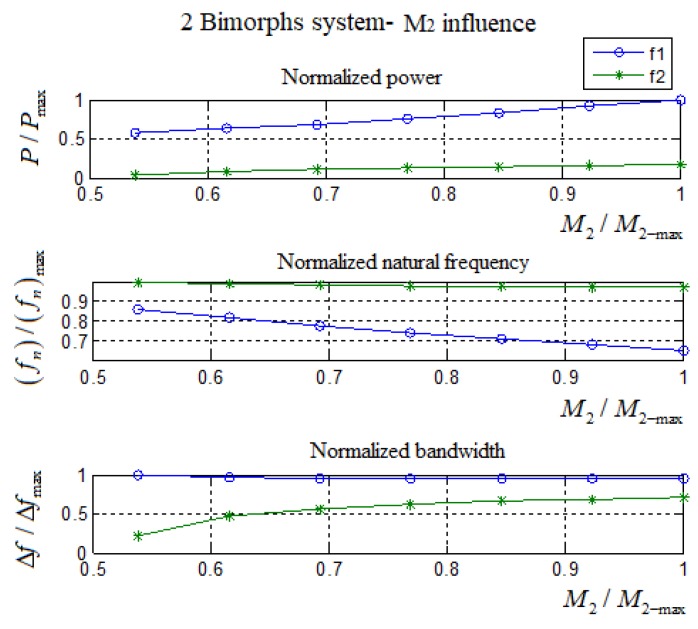
The influence of the normalized *M*_2_ mass on the output power, the natural frequencies, and the bandwidth (all normalized)—two bimorphs interconnected by a spring case.

**Figure A6:**
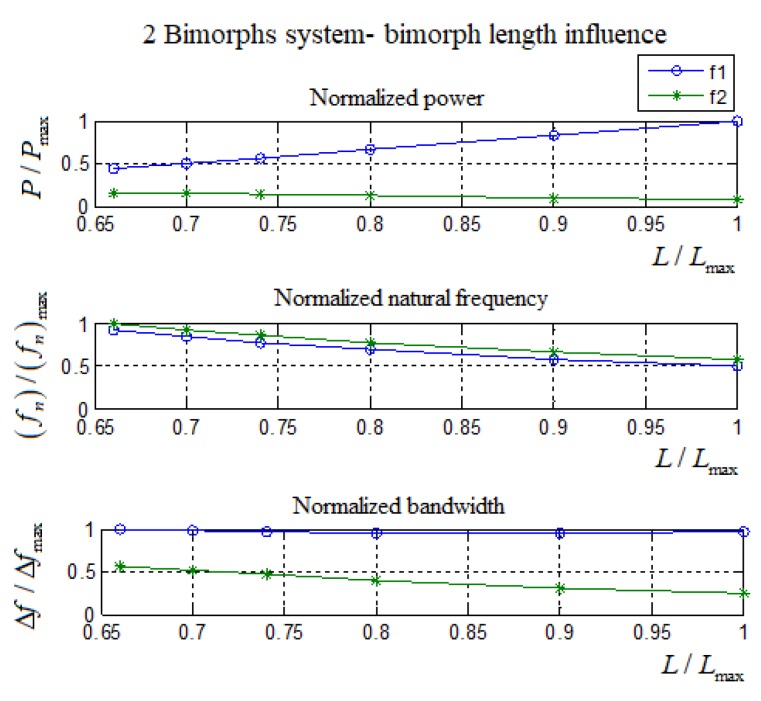
The influence of the normalized bimorph length on the output harvested power, natural frequency, and bandwidth (all normalized)—two bimorphs interconnected by a spring case.

**Figure A7:**
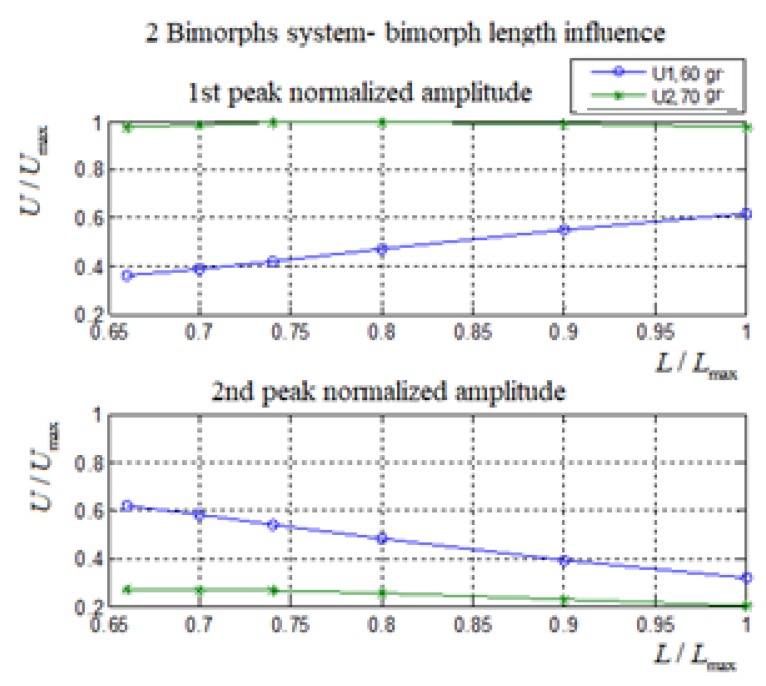
The influence of the normalized bimorph length on the normalized amplitude at the first and second peaks—two bimorphs interconnected by a spring case.

**Figure A8:**
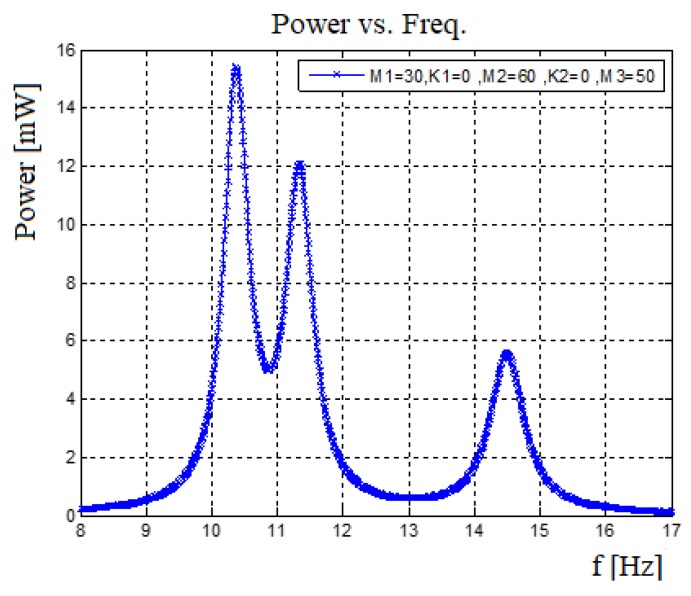
Power output for no spring connections (*K*_1_ = *K*_2_ = 0)—three bimorphs interconnected by two springs case.

**Figure A9:**
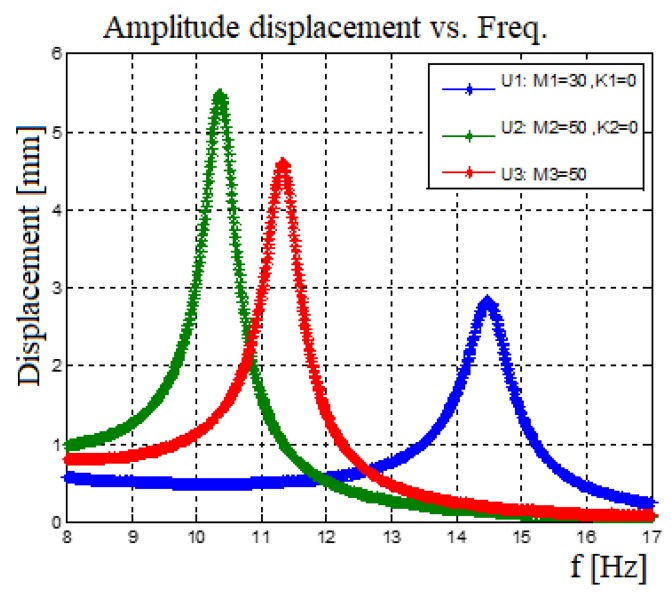
Amplitude displacement for no spring connections (*K*_1_ = *K*_2_ = 0)—three bimorphs interconnected by two springs case.

**Figure A10:**
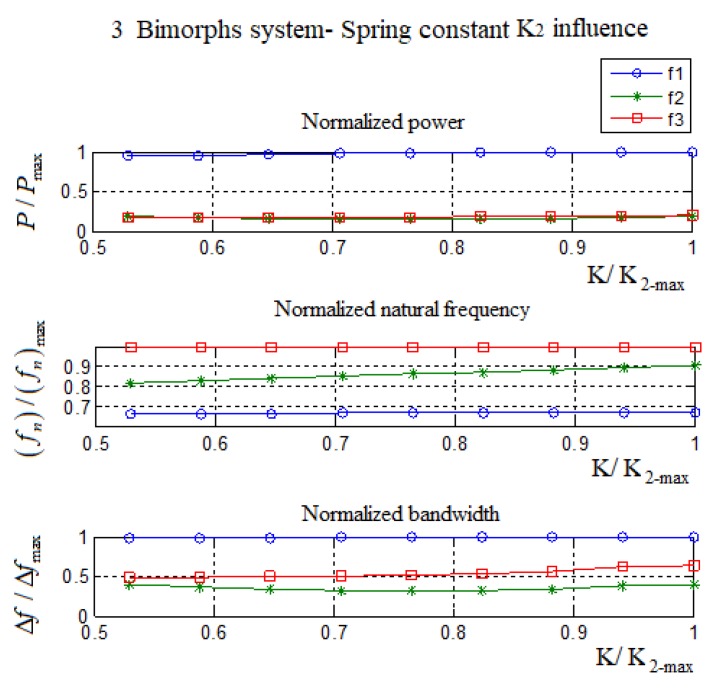
The influence of the normalized spring constant, *K*_2_ on the output harvested power, natural frequency, and bandwidth (all normalized)—three bimorphs interconnected by two springs case.

**Figure A11:**
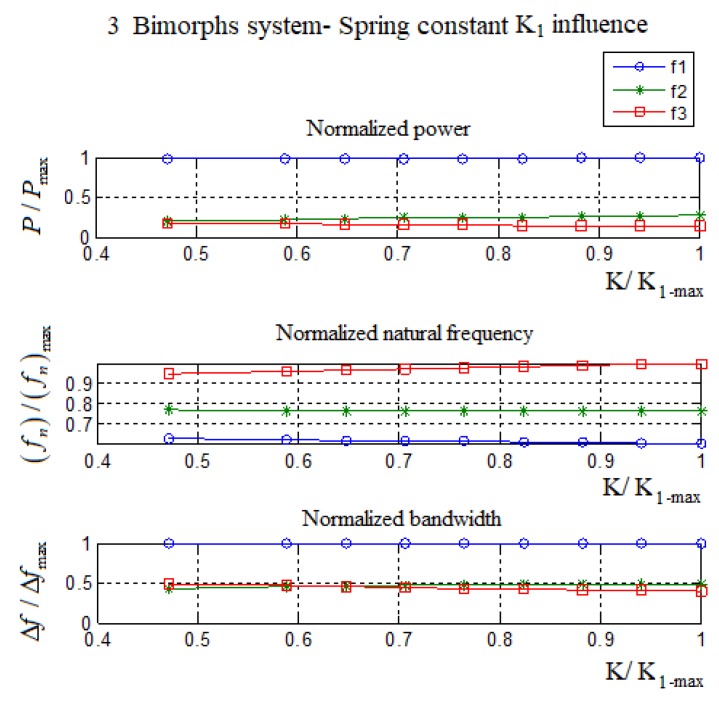
The influence of the normalized spring constant, *K*_1_ on the output harvested power, natural frequency, and bandwidth (all normalized)—three bimorphs interconnected by two springs case.

## Appendix A. Derivation of the Electric Power for a Bimorph under Vibration Excitation

The piezoelectric bimorph, shown schematically in Figure A1, consists of a pair of identical piezoelectric twin layers poled along the thickness direction and separated by a metallic carrying beam in the middle. The bimorph is a cantilever beam, with the clamped side being excited harmonically in the vertical direction (*z*) with a known amplitude, A, and a given excitation frequency, ω. At the free side of the cantilever beam, a mass M_0_ is connected. As shown in Figure A1, the piezoelectric electrodes are connected in parallel to an external impedance *Z_L_*.

Assuming that the bimorph is a slender beam, we can write the following piezoelectric equations for the present case (where the *x, y, z* system is the usual 1, 2, and 3):

(A1) $$\begin{array}{l} S_{x}=s_{11}T_{x}+d_{31}E_{z}\\ D_{z}=d_{31}T_{x}+\varepsilon_{33}E_{z} \end{array}\;$$

where S_x_ and T_x_ are the strain and the stress in the x direction (along the beam-Figure A1), respectively, D_3_ is the electrical displacement, and E_z_ is the electrical field induced due to the vibrations. The constants, s_11_, d_31_, and ε_33_ are the axial elastic compliance measured at constant electric field, the transverse piezoelectric constant, and the transverse dielectric constant measured at fixed stress, respectively. For the present case, we have the following expressions for the strain and the electric field:

(A2) $$\begin{array}{l} S_{x}=-zw{,}_{xx}\\ E_{z}=-V/h \end{array}\;$$

where w is the transverse displacement, V is the voltage accumulated on the piezoelectric electrodes due to the induced vibrations, and h is the thickness of piezoelectric layer. Solving for T_x_ from the first equation in Equation (A1), and then substituting into the second one yielding D_z_, we get

(A3) $$\begin{array}{l} T_{x}=s_{11}^{-1}S_{x}-s_{11}^{-1}d_{31}E_{z}\\ D_{z}=s_{11}^{-1}d_{31}S_{x}+{\bar{\varepsilon }}_{33}E_{z} \end{array}\;$$

where

(A4) $$\begin{array}{l} {\bar{\varepsilon }}_{33}=\varepsilon_{33}\left(1-k_{31}^{2}\right)k_{31}^{2}=\frac{d_{31}^{2}}{\varepsilon_{33}\times s_{11}} \end{array}\;$$

One should note that the stress in the middle layer (the metal layer) is given by

(A5) $$T_{x}=s_{11}^{-1}S_{x}=E_{substrate}S_{x}=-E_{substrate}zw{,}_{xx}\;$$

where E_substrate_ is the Young’s modulus of the metallic layer. The bending moment is given by

(A6) $$M=\int zT_{x}dydz=-Dw{,}_{xx}+s_{11}^{-1}\times d_{31}\times \frac{V}{h}\times \left(H+h\right)\times h\times b=-Dw{,}_{xx}+E_{piezo}\times d_{31}\times V\times \left(H+h\right)\times b\;$$

with

(A7) $$D=\left\{\frac{2}{3}\times E_{substrate}\times {\left(\frac{H}{2}\right)}^{3}+\frac{2}{3}\times E_{piezo}\left[{\left(\frac{H}{2}+h\right)}^{3}-{\left(\frac{H}{2}\right)}^{3}\right]\right\}\times b\;$$

The shear force in the beam is given by the differentiation of the moment, namely

(A8) $$\overset{⌣}{V}=-M,x=+D\times w{,}_{xxx}\;$$

The flexural vibration of the slender bimorph beam is then written as

(A9) $$M{,}_{xx}=m\ddot{w}\begin{array}{ccc} & \Rightarrow & -D\times w{,}_{xxxx}= \end{array}m\ddot{w}\;$$

with the mass per unit length being given by the following expression

(A10) $$m=H\times b\times \rho_{Al}+2\times h\times b\times \rho_{piezo}\;$$

Let us calculate the electric charge accumulated on the top electrode at z = (h + H/2); integrating the electrical displacement on the relevant surface gives

(A11) $$\begin{array}{l} Q_{top}=-\int_{0}^{L}\int_{0}^{b}D_{z}{}_{@\left(z=\frac{H}{2}+h\right)}dx\times dy=\\ =-\left\{b\times E_{piezo}\times d_{31}\left(\frac{H}{2}+h\right)\left[w{,}_{x}\left(L,t\right)-w{,}_{x}\left(L,t\right)\right]+b\times {\bar{\varepsilon }}_{33}\frac{V}{h}L\right\} \end{array}\;$$

The relation between the charge and the current is known to be as

(A12) $$I=\frac{dQ}{dt}\;$$

which leads for our case to the relation of the voltage as a function of the two piezoelectric layers.

(A13) $$2I=\frac{\hat{V}}{z_{L}}\begin{array}{ccc} & \Rightarrow & \hat{V}=2\times I\times z_{L} \end{array}\;$$

The boundary conditions of the cantilever beam, having a piezoelectric bimorph can be written as

(A14) $$@x=0\begin{array}{cc} & w\left(0,t\right)=Ae^{i\omega t} \end{array}\begin{array}{cc} ; & w{,}_{x}\left(0,t\right)=0. \end{array}\;$$

(A15) $$@x=L\begin{array}{cc} & M\left(L,t\right)=0 \end{array}\begin{array}{cc} ; & \overset{⌣}{V}\left(L,t\right)=M_{0}\frac{\partial^{2}w\left(L,t\right)}{\partial t^{2}} \end{array}\;$$

Using Equation (A9), together with the boundary conditions given by Equations (A14) and (A15), we can suggest a solution for the posed problem, having the following form:

(A16) $$\left\{\begin{array}{c} w(x,t)\\ \hat{V}(t)\\ Q\left(t\right)\\ I\left(t\right) \end{array}\right\}=\left\{\begin{array}{c} W(x)\\ \tilde{V}\\ \tilde{Q}\\ \tilde{I} \end{array}\right\}e^{{}^{i\omega t}}\;$$

Substituting the proposed solution into Equation (A9) and the associated boundary conditions yields

(A17) $$D\frac{d^{4}W}{dx^{4}}-m\omega^{2}W=0\;$$

The solution of Equation (A17) has the following form:

(A18) $$W=A_{1}\cosh(\beta x)+A_{2}\sinh(\beta x)+A_{3}\cos(\beta x)+A_{4}\sin(\beta x)\;$$

with the *A*_1_–*A*_4_ coefficients to be determined from the boundary conditions and

(A19) $$\beta^{4}\equiv \frac{m\omega^{2}}{D}\;$$

The associated boundary conditions presented by Equations (A14) and (A15) transform into the following expressions:

(A20) $$@x=0\begin{array}{cc} & W\left(0\right)=A \end{array}\begin{array}{cc} ; & W{,}_{x}\left(0\right)=0 \end{array}\;$$

(A21) $$\begin{array}{l} @x=L\begin{array}{cc} & -D\cdot W{,}_{xx}(L)+E_{piezo}d_{31}\frac{\tilde{V}}{h}\times \left(H+h\right)\times h\times b=0 \end{array};\\ \begin{array}{cccc} & & & \begin{array}{cc} & D\cdot W{,}_{xxx}(L)=-M_{0}\times \omega^{2}\times W(L) \end{array} \end{array} \end{array}\;$$

The expression for the current will have the following form:

(A22) $$\begin{array}{l} \tilde{I}=i\times \omega \times b\cdot \\ \left\{E_{piezo}\times d_{31}\left(\frac{H}{2}+h\right)\times \beta \times \left[-A_{1}\sinh\left(\beta L\right)-A_{2}\cosh\left(\beta L\right)+A_{3}\sin\left(\beta L\right)-A_{4}\cos\left(\beta L\right)\right]-{\bar{\varepsilon }}_{33}\frac{\tilde{V}}{h}L\right\} \end{array}\;$$

Applying the boundary conditions (Equations (A20) and (A21)) we get the four equations for the four constants *A*_1_–*A*_4_.

(A22) $$A_{1}+A_{3}=A\;$$

(A23) $$A_{2}\beta +A_{4}\beta =0\;$$

(A24) $$\begin{array}{l} -D\times \left[A_{1}\times \beta^{2}\times \cosh(\beta L)+A_{2}\times \beta^{2}\times \sinh(\beta L)-A_{3}\times \beta^{2}\times \cos(\beta L)-A_{4}\times \beta^{2}\times \sin(\beta L)\right]\\ +E_{piezo}d_{31}\times \tilde{V}\times \left(H+h\right)\times b=0 \end{array}\;$$

(A25) $$\begin{array}{l} D\times \left[A_{1}\times \beta^{3}\times \sinh(\beta L)+A_{2}\times \beta^{3}\times \cosh(\beta L)+A_{3}\times \beta^{3}\times \sin(\beta L)-A_{4}\times \beta^{3}\times \cos(\beta L)\right]\\ =-M_{0}\times \omega^{2}\times \left[A_{1}\times \cosh(\beta L)+A_{2}\times \sinh(\beta L)+A_{3}\times \cos(\beta L)+A_{4}\times \sin(\beta L)\right] \end{array}\;$$

plus an expression for the voltage generated

(A26) $$\begin{array}{l} \frac{\tilde{V}}{2\times z_{L}\times i\times \omega \times b}=E_{piezo}\times d_{31}\left(\frac{H}{2}+h\right)\times \beta \times \left[-A_{1}\sinh\left(\beta L\right)-A_{2}\cosh\left(\beta L\right)+A_{3}\sin\left(\beta L\right)-A_{4}\cos\left(\beta L\right)\right]\\ -{\bar{\varepsilon }}_{33}\frac{\tilde{V}}{h}L \end{array}\;$$

or

(A27) $$\tilde{V}\times \left(\frac{1}{2z_{L}}+\frac{1}{z_{0}}\right)=E_{piezo}\times d_{31}\left(\frac{H}{2}+h\right)\times \beta \times \left[-A_{1}\sinh\left(\beta L\right)-A_{2}\cosh\left(\beta L\right)+A_{3}\sin\left(\beta L\right)-A_{4}\cos\left(\beta L\right)\right]\;$$

where

(A28) $$C_{0}=\frac{{\bar{\varepsilon }}_{33}\times b\times L}{h};\begin{array}{cc} & z_{0}=\frac{1}{i\times \omega \times C_{0}} \end{array}\;$$

Note that the impedances of $z_{L}$ and $z_{0}$ depend on the input excitation frequency ω, whose specific form depends on the specific structure of the output circuit. The explicit expressions for the four constants and the voltage are next presented.

(A29) $$\begin{array}{l} A_{1}=\frac{A}{\Delta }[-2\times M_{0}\times \omega^{2}\times D\times \beta \times \cos\left(\beta \times L\right)\times \sinh\left(\beta \times L\right)-D\times \beta^{4}\times \cos^{2}\left(\beta \times L\right)]+\\ \begin{array}{cc} & M_{0}\times \omega^{2}\times \delta \times \left[1-\cos\left(\beta \times L\right)\times \cosh\left(\beta \times L\right)-\sin\left(\beta \times L\right)\times \sinh\left(\beta \times L\right)\right]+ \end{array}\\ \begin{array}{cc} & \begin{array}{l} -D^{2}\times \beta^{4}\times \left[\sin^{2}\left(\beta \times L\right)+\cos\left(\beta \times L\right)\times \cosh\left(\beta \times L\right)+\sin\left(\beta \times L\right)\times \sinh\left(\beta \times L\right)\right]+\\ -D\times \beta^{3}\times \delta \times \sin\left(\beta \times L\right)\times \cosh\left(\beta \times L\right)-\beta^{3}\times \delta \times \sin\left(\beta \times L\right)\times \cos\left(\beta \times L\right)] \end{array} \end{array} \end{array}\;$$

(A30) $$\begin{array}{l} A_{2}=\frac{A}{\Delta }[2\times M_{0}\times \omega^{2}\times D\times \beta \times \cos\left(\beta \times L\right)\times \sinh\left(\beta \times L\right)-\beta^{4}\times \sin^{2}\left(\beta \times L\right)+\\ M_{0}\times \omega^{2}\times \delta \times \left[\sin\left(\beta \times L\right)\times \cosh\left(\beta \times L\right)+\cos\left(\beta \times L\right)\times \sinh\left(\beta \times L\right)\right]+\\ D^{2}\times \beta^{4}\times \left[\cos\left(\beta \times L\right)\times \sinh\left(\beta \times L\right)+\cosh\left(\beta \times L\right)\times \sin\left(\beta \times L\right)+\cos\left(\beta \times L\right)\times \sin\left(\beta \times L\right)\right]+\\ D\times \beta^{3}\times \delta \times \left[2\times \sin\left(\beta \times L\right)\times \sinh\left(\beta \times L\right)+\sin^{2}\left(\beta \times L\right)\right]-D\times \beta^{4}\times \delta \times \sin\left(\beta \times L\right)\times \cos\left(\beta \times L\right)] \end{array}\;$$

(A31) $$\begin{array}{l} A_{3}=A-A_{1}=A-\frac{A}{\Delta }[-2\times M_{0}\times \omega^{2}\times D\times \beta \times \cos\left(\beta \times L\right)\times \sinh\left(\beta \times L\right)-D\times \beta^{4}\times \cos^{2}\left(\beta \times L\right)]\\ \begin{array}{cc} & +M_{0}\times \omega^{2}\times \delta \times \left[1-\cos\left(\beta \times L\right)\times \cosh\left(\beta \times L\right)-\sin\left(\beta \times L\right)\times \sinh\left(\beta \times L\right)\right]+ \end{array}\\ \begin{array}{cc} & \begin{array}{l} -D^{2}\times \beta^{4}\times \left[\sin^{2}\left(\beta \times L\right)+\cos\left(\beta \times L\right)\times \cosh\left(\beta \times L\right)+\sin\left(\beta \times L\right)\times \sinh\left(\beta \times L\right)\right]+\\ -D\times \beta^{3}\times \delta \times \sin\left(\beta \times L\right)\times \cosh\left(\beta \times L\right)-\beta^{3}\times \delta \times \sin\left(\beta \times L\right)\times \cos\left(\beta \times L\right)] \end{array} \end{array} \end{array}\;$$

(A32) $$\begin{array}{l} A_{4}=-A_{2}=-\frac{A}{\Delta }[2\times M_{0}\times \omega^{2}\times D\times \beta \times \cos\left(\beta \times L\right)\times \sinh\left(\beta \times L\right)-\beta^{4}\times \sin^{2}\left(\beta \times L\right)]+\\ M_{0}\times \omega^{2}\times \delta \times \left[\sin\left(\beta \times L\right)\times \cosh\left(\beta \times L\right)+\cos\left(\beta \times L\right)\times \sinh\left(\beta \times L\right)\right]+\\ D^{2}\times \beta^{4}\times \left[\cos\left(\beta \times L\right)\times \sinh\left(\beta \times L\right)+\cosh\left(\beta \times L\right)\times \sin\left(\beta \times L\right)+\cos\left(\beta \times L\right)\times \sin\left(\beta \times L\right)\right]+\\ D\times \beta^{3}\times \delta \times \left[2\times \sin\left(\beta \times L\right)\times \sinh\left(\beta \times L\right)+\sin^{2}\left(\beta \times L\right)\right]-D\times \beta^{4}\times \delta \times \sin\left(\beta \times L\right)\times \cos\left(\beta \times L\right)] \end{array}\;$$

and

(A33) $$\begin{array}{l} \tilde{V}=\frac{\gamma }{\alpha }\left(\frac{H}{2}+h\right)\times \beta \times \left[-A_{1}\sinh\left(\beta L\right)-A_{2}\cosh\left(\beta L\right)+\left(A-A_{1}\right)\sin\left(\beta L\right)+A_{2}\cos\left(\beta L\right)\right] \end{array}\;$$

where the constants A_1_–A_4_ are defined in Equations (30A)–(33A) and Δ is defined as

(A34) $$\begin{array}{l} \Delta =-2\times \left(D^{2}\times \beta^{4}-M_{0}\times \omega^{2}\times \delta \right)\cosh\left(\beta \times L\right)\times \cos\left(\beta \times L\right)-2\times D^{2}\times \beta^{4}+M_{0}\times \omega^{2}\times \delta \\ \begin{array}{cc} & - \end{array}\left(D^{2}\times \beta^{3}\times \delta -\beta^{3}\times \delta -2\times M_{0}\times \omega^{2}\times \beta \times D\right)\times \sinh\left(\beta \times L\right)\times \sin\left(\beta \times L\right)\\ \begin{array}{cc} & - \end{array}\left(D^{2}\times \beta^{3}\times \delta -\beta^{3}\times \delta \right)\times \sin\left(\beta \times L\right)\times \cosh\left(\beta \times L\right)+2\times M_{0}\times \omega^{2}\times \delta \times \beta \times D \end{array}\;$$

with

(A35) $$\begin{array}{l} \alpha =\left(\frac{1}{2\times z_{L}}+\frac{1}{z_{0}}\right);\begin{array}{cc} & \gamma =E_{piezo}\times d_{31};\begin{array}{cc} & \delta =\frac{\gamma^{2}}{\alpha } \end{array} \end{array}\times \left(H+h\right)\times \left(\frac{H}{2}+h\right)\times b \end{array}\;$$

Having the explicit expressions, one can calculate the behavior of the vibrating bimorph for various input amplitude, A, and excitation frequency, ω, with the external impedance $z_{L}$ being a parameter.

## Appendix B. Bimorph Series and Parallel Electrical Connections

The two types of electrical connections used for bimorph are schematically presented in Figure A2.

## Appendix C. The Data Used for the Comparison with the Results in [24]

The data used in Ref. [24] was the following one:

PZT 5H: $s_{11}=16.5\times 10^{-12}m^{2}/N$, $\;d_{31}=-274\times 10^{-12}C/N$, $\;\varepsilon_{33}=3400\varepsilon_{0}$, $\rho =7500\mathrm{kg}/m^{3}$;

Quality factor: $Q=10^{2}$;

Bimorph dimensions: L=25 mm,b=8 mm,h=2 mm,c=2 mm;

The acceleration amplitude: $\omega^{2}A=1m/s^{2}$ ;

For the elastic substrate: $E=70\;\mathrm{GPa},\rho =2700\mathrm{kg}/m^{3}$.

The spring constant and the relative impedance are defined as

(C1) $$K_{0}=\frac{3EI}{L^{3}};\begin{array}{cc} & Z_{0}=\frac{1}{i\omega C_{0}} \end{array};\begin{array}{cc} & Z_{L} \end{array}=iZ_{0}\;$$

where

(C2) $$I_{0}=\frac{b{\left(2h+2c\right)}^{3}}{12};\begin{array}{cc} & C_{0}=\frac{\varepsilon_{33}bL}{h} \end{array}\;$$

## Appendix D. Two Bimorphs System-Further Parametric Investigations

### Appendix D.1. The Influence of the Connecting Spring Constant 3 Parameters were Chosen to Be Investigated

- First and second natural frequencies of the system;
- Peak power (at the natural frequencies);
- Bandwidth, Δf, for each natural frequency.

A common definition of bandwidth involves finding the area where the amplitude is above $A_{max}/\sqrt{2}$, where $A_{max}$ is the maximum amplitude. Here, the bandwidth was chosen by the area where the power is above 1 (mW) for the first natural frequency and above 0.5 mW for the second natural frequency. The influence of the spring constant on the system’s performance is presented in Table A1.

**Table A1:** Two bimorphs interconnected by a spring system—the influence of the spring constant on the harvested power.

f Hz	f Hz					
	f Hz		Power mW		Δf Hz	
	1st Peak	2nd Peak	1st Peak	2nd Peak	1st Peak	2nd Peak
40	11.91	15.77	10.33	1.832	1.81	1.19
60	12.05	16.47	10.85	1.33	1.88	0.87
80	12.15	17.17	11.11	1.001	1.93	0.63
100	12.23	17.86	11.23	0.7801	1.96	0.44
120	12.28	18.53	11.30	0.6255	1.99	0.27
150	12.34	19.52	11.33	0.4697	2.01	0.22 *

* The bandwidth for 150 N/m 2nd peak, is at the region where the power is above 0.4 mW contrary to the other values of 0.5 mW due to low power for this spring constant.

To further understand the influence of the spring constant, all the values in Table A3 were normalized and presented in Table A2 and Figure A3. The normalization has been done separately for the power, natural frequency, and bandwidth by dividing each parameter by its maximum value. In Figure A3, the *X* axis is the normalized spring constant (the spring constant divided by the maximum spring constant = 150 N/m).

**Table A2:** Two bimorphs connected by a spring system—the influence of the normalized spring constant on the normalized harvested power.

$\;K/K_{max}\;$	$\;f_{n}/f_{n}{}_{max}\;$		$\;P/P_{max}\;$		$\;\Delta f/\Delta f_{max}\;$	
	1st Peak	2nd Peak	1st Peak	2nd Peak	1st Peak	2nd Peak
0.267	0.610	0.808	0.912	0.162	0.900	0.592
0.400	0.617	0.844	0.958	0.117	0.935	0.433
0.533	0.622	0.880	0.981	0.088	0.960	0.313
0.667	0.627	0.915	0.991	0.069	0.975	0.219
0.800	0.629	0.949	0.997	0.055	0.990	0.134
1.000	0.632	1.000	1.000	0.041	1.000	0.109

From Table A3 and Table A4 and Figure A3, it can be clearly observed that as the spring gets stiffer (increasing the value of its constant), the second natural frequency increases, while the harvested power at this natural frequency gets smaller, tending to negligible values. Stiffening the spring connecting the two bimorphs leads to a small rise in the first natural frequency of the system, yielding larger values for the harvested power at this frequency. The increase in the output harvested power (at the first frequency) with the stiffening of the spring can be attributed to the movement of the two bimorphs and is dictated by the heavier mass, which causes the lighter mass to vibrate at a larger amplitude, leading to a higher output power. At a given value of the nondimensional spring, its influence is reduced, and instead of two masses, we obtain a single mass (the sum of the two) at the end of a single beam made of two beams connected by a stiff spring (which do not allow relative beams movements). At the second system natural frequency, the stiffening of the spring causes the second frequency to increase while the generated power and bandwidth decrease. As the spring constant becomes larger in comparison with the stiffness of the vibrating beams, we get, as before, a single beam while the second natural frequency is diminishing, yielding a single frequency attributed to the division of the equivalent stiffness to the total end masses.

### Appendix D.2. The Influence of the End Mass

Increasing the mass ratio in the parametric simulation was done by increasing the second bimorph mass, *M*_2_, while the first end mass, *M*_1_, remains constant- 30 gr.

First it is interesting to browse at Figure A4, displaying that two different end masses would yield a better harvester than having the same mass, as the systems’ bandwidth would increase.

Note that the parameters chosen to be investigated are the same as for the influence of spring constant, natural frequencies, output power and frequency bandwidth. The influence of the M_2_ mass value on the output harvested power is summarized in Table A3.

**Table A3:** Two bimorphs interconnected by a spring system—the influence of the value of *M*_2_ on the harvested power (*M*_1_ is constant_1_ = 30 gr).

*M*_2_ gr	f Hz		Power mW		Δ*f* Hz	
	1st Peak	2nd Peak	1st Peak	2nd Peak	1st Peak	2nd Peak
30	14.49	-	7.315	-	1.92	-
35	13.83	16.11	7.988	0.6114	1.91	0.41
40	13.13	15.93	8.614	1.156	1.85	0.89
45	12.48	15.83	9.398	1.559	1.82	1.08
50	11.91	15.77	10.33	1.832	1.81	1.19
55	11.4	15.73	11.38	2.023	1.81	1.26
60	10.94	15.7	12.53	2.161	1.82	1.31
65	10.53	15.69	13.74	2.266	1.82	1,34

To further understand the influence of the value of mass *M*_2_, all the values in Table A3 were normalized and presented in Table A4 and Figure A5, in which the X axis is the mass normalized by the maximal *M*_2_ = 65 gr.

**Table A4:** Two bimorphs interconnected by a spring system—the influence of the normalized *M*_2_ on the normalized natural frequencies, harvested power, and bandwidth.

*M*_2_/*M*_2_max_ *	$\;f_{n}/f_{n}{}_{max}$		$\;P/P_{max}$		$\;\Delta f/\Delta f_{max}$	
	1st Peak	2nd Peak	1st Peak	2nd Peak	1st Peak	2nd Peak
0.54	0.858	1.000	0.581	0.044	1.00	0.21
0.62	0.815	0.989	0.627	0.084	0.97	0.47
0.69	0.775	0.983	0.684	0.113	0.95	0.57
0.77	0.739	0.979	0.752	0.133	0.95	0.62
0.85	0.708	0.976	0.828	0.147	0.95	0.66
0.92	0.679	0.975	0.912	0.157	0.95	0.69
1.000	0.654	0.974	1.000	0.165	0.95	0.7

* M_2-max_=65 gr.

Table A4 and Figure A5 clearly present the following behavior with an increase in the end mass ratio:

- A slight reduction of the first natural frequency leads to an increase in the harvested power;
- In parallel, the second natural frequency is reducing while the harvested power increases.

Remembering the known expression for the natural frequency (ω) $=\sqrt{\frac{K}{M}}$, shows that as the mass gets larger, the natural frequency is reduced. This explains why the natural frequencies get lower with the increase of the mass ratio. However, in contrary to the behavior of the natural frequency, the harvested power increases. Regarding the end mass amplitudes (see Figure 20), one can observe that the amplitude increases when the natural frequency decreases (end mass ratio increase). As the analytic model, developed within the present research, assumes a constant displacement amplitude, namely $=\frac{Const}{\omega^{2}}$, it is expected that the amplitude increases for a decrease in the input excitation frequency. Also, as expected, the “power pit” is narrowing, yielding a narrow frequency bandwidth, as the values of the vibrating end masses are getting closer.

### Appendix D.3. The Influence of the Bimorph Length

The influence of the length (L) of the bimorphs on the output harvested power and the relevant end mass amplitude and phase was next investigated. The end masses used for the parametric investigation were: *M*_1_ = 60 gr and *M*_2_ = 70 gr. The spring constant was taken as *K* = 40 N/m. The influence of the length of the bimorphs on the performance of the present harvester is summarized in Table A5, while its influence on the end mass amplitude is summarized in Table A6 with U_1_ being the amplitude of *M*_1_ end mass and *U*_2_ is the amplitude of *M*_2_ end mass.

**Table A5:** The influence of the bimorph length on the output harvested power—two bimorphs interconnected by a spring case.

	*M*_1_ = 60 gr, *M*_2_ = 70 gr, *K* = 40 N/m					
*L* mm	*f* Hz		Power mW		Δf Hz	
	1st Peak	2nd Peak	1st Peak	2nd Peak	1st Peak	2nd Peak
33	18.28	19.91	9.61	3.31	2.54	1.42
35	16.76	18.33	10.86	3.217	2.49	1.29
37	15.44	16.97	12.17	3.07	2.44	1.18
40	13.76	15.27	14.24	2.776	2.41	1.01
45	11.56	13.1	17.85	2.208	2.41	0.79
50	9.88	11.52	21.55	1.679	2.44	0.62

**Table A6:** The influence of the bimorph length on the harvester amplitude—two bimorphs interconnected by a spring case.

*L* mm	M_1_ = 60 gr, M_2_ = 70 gr, *K* = 40 N/m							
	1st Peak				2nd Peak			
	$f_{n}\;\;\mathrm{Hz}$	U_1_ mm	U_2_ mm	Power mW	$f_{n}\;\;\mathrm{Hz}$	*U*_1_ mm	*U*_2_ mm	Power mW
33	18.28	8.202	22.17	9.61	19.91	13.97	6.107	3.31
35	16.76	8.808	22.41	10.86	18.33	13.12	6.042	3.217
37	15.44	9.504	22.57	12.17	16.97	12.23	5.97	3.07
40	13.76	10.64	22.66	14.24	15.27	10.91	5.735	2.776
45	11.56	12.48	22.49	17.85	13.1	8.86	5.228	2.208
50	9.88	14.06	22.07	21.55	11.52	7.202	4.58	1.679

For design purposes, the values in Table A5 are normalized and presented in Table A7 and Figure A6. The amplitude normalized values are presented in Table A8 and Figure A7. The length values are normalized by the maximum length $L_{max}=$ 50 mm.

**Table A7:** The influence of the normalized bimorph length on the normalized output harvested power—two bimorphs interconnected by a spring case.

L/L__max_	$\;f_{n}/f_{n}{}_{max}$		$\;P/P_{max}$		$\;\Delta f/\Delta f_{max}$	
	1st Peak	2nd Peak	1st Peak	2nd Peak	1st Peak	2nd Peak
0.66	0.92	1.00	0.45	0.15	1.00	0.56
0.7	0.84	0.92	0.5	0.15	0.98	0.51
0.74	0.78	0.85	0.56	0.14	0.96	0.46
0.8	0.69	0.77	0.66	0.13	0.95	0.4
0.9	0.58	0.66	0.83	0.1	0.95	0.31
1.00	0.5	0.58	1.00	0.08	0.96	0.24

**Table A8:** The influence of the normalized bimorph length on the normalized end mass amplitude—two bimorphs interconnected by a spring case.

*L*/*L*_max	*U*_1_/Umax		*U*_2_/Umax	
	1st Peak	2nd Peak	1st Peak	2nd Peak
0.66	0.362	0.978	0.617	0.27
0.7	0.389	0.989	0.579	0.267
0.74	0.419	0.996	0.54	0.263
0.8	0.47	1.000	0.481	0.253
0.9	0.551	0.992	0.391	0.231
1.00	0.62	0.974	0.318	0.202

As can be seen from Table A7 and Figure A6, increasing the bimorphs length leads to the decrease of the natural frequencies and the bandwidth, while the harvested power at the first natural frequency increases and at second natural frequency slightly decreases. It is interesting to note that an increase in the length of the bimorphs does not alter the distance between the first two natural frequencies and only shifts them to the left. This phenomenon might be well exploited by the designer if the harvester has the ability to extend or shorten the bimorphs length as a function of the excitation input frequency.

Figure A7 shows that at the first natural frequency the amplitude of the lower mass increases with the bimorphs length increase while the amplitude of the higher mass is almost not changing. This can be explained by the relative stiffness of the spring to the stiffness of the bimorphs. As the bimorphs length increase the stiffness of the bimorphs compare to the connection spring is getting weaker so the influence of spring is coming more into account. Thus, the lower mass bimorph is pushed more and more by the higher mass (the spring acts like a stiff bar). For the second natural frequency, the explanation is same, but in this case, the spring resonates the two bimorphs and thus their amplitude decreases even to negligible values.

Additional parameters results, which were also parametrically investigated—like the influence of the width of the bimorph, the influence of the substrate thickness, and the influence of the piezo-layer thickness on the generated power—can be found in [43].

## Appendix E. Three Bimorphs System-Further Parametric Investigations

### Appendix E.1. Spring Constant (K_2_) Connected to the External Larger Mass Influence

First, we shall examine the case of no springs at all (*K*_1_ = *K*_2_ = 0). The generated power and the experienced displacements of the three bimorphs are presented in Figure A8 and Figure A9, respectively. The power output is the sum of the three bimorphs individual outputs and there is no mutual influence among the three bimorphs.

The influence of the spring *K*_2_ constant connecting the middle mass to the heavier external mass was next parametrically investigated with the end masses having the values of *M*_1_ = 30 gr, *M*_2_ = 60 gr, and *M*_3_ = 50 gr, the constant spring is *K*_1_ = 40 N/m, while the value of *K*_2_ constant is varied (the bimorphs were kept identical as before, with *L* = 50 mm, *b* = 30 mm, *c* = 0.1 mm). Normalized values of the parameters are presented in Table A9 and Figure A10 with *K*_2_ values being normalized by *K*_2-max_ = 85 N/m.

**Table A9:** Three bimorphs system—influence of K_2_ spring constant connected to the external larger mass-normalized values

*K*_2_/*K*_2-max_	$\;f_{n}/f_{n}{}_{max}$			$\;P/P_{max}$			$\;\Delta f/\Delta f_{max}$		
	1st Peak	2nd Peak	3rd Peak	1st Peak	2nd Peak	3rd Peak	1st Peak	2nd Peak	3rd Peak
0.529	0.662	0.812	1.000	0.941	0.18	0.169	0.977	0.391	0.482
0.588	0.664	0.831	1.000	0.955	0.169	0.17	0.982	0.359	0.491
0.647	0.666	0.842	0.999	0.966	0.16	0.172	0.982	0.341	0.496
0.706	0.666	0.852	0.999	0.975	0.155	0.175	0.986	0.327	0.505
0.765	0.668	0.863	0.998	0.982	0.152	0.178	0.986	0.323	0.514
0.824	0.668	0.874	0.998	0.988	0.153	0.181	0.996	0.323	0.532
0.882	0.669	0.885	0.997	0.993	0.158	0.186	0.996	0.332	0.559
0.941	0.67	0.896	0.997	0.997	0.167	0.191	1.000	0.386	0.623
1.000	0.67	0.907	0.996	1.000	0.186	0.199	1.000	0.4	0.627

From Figure A10 and Table A9 it can be observed that as the value of *K*_2_ spring increases (the spring connecting the middle mass to the external larger mass) the second natural frequency increases while the first and third ones are almost constant. Thus, the “power pit” between the first and second natural frequencies increases while the “power pit” between the second and third natural frequencies decreases. The power of each bimorph is almost constant with the shift of the second natural frequency, and the same thing is true also for the bandwidth. This increase of the second natural frequency can be attributed to *K*_2_ constant increase, the 2nd natural frequency, for which the larger external mass is “responsible”, shifts to right side of the graph, and the generated power would decrease (see the case for the influence of the spring constant for a two bimorphs system).

### Appendix E.2. Spring Constant (K_1_) Connected to the External Lighter Mass Influence

The influence of the spring constant (*K*_1_) connecting the middle mass to the lighter external mass, was then investigated and the respective results are next presented in Table A10 and Figure A11. All the values used in the previous subsection were kept, while the value of the spring *K*_2_ constant is *K*_2_ = 40 N/m. The constant spring *K*_1_ was normalized by *K*_1-max_ = 85 N/m.

**Table A10:** Three bimorphs system—influence of *K*_1_ spring constant connected to the external lighter mass normalized values.

*K*_1_/*K*_1_-max	$\;f_{n}/f_{n}{}_{max}$			$\;P/P_{max}$			$\;\Delta f/\Delta f_{max}$		
	1st Peak	2nd Peak	3rd Peak	1st Peak	2nd Peak	3rd Peak	1st Peak	2nd Peak	3rd Peak
0.471	0.627	0.768	0.949	0.973	0.205	0.176	0.995	0.433	0.488
0.588	0.621	0.767	0.961	0.971	0.223	0.166	0.995	0.451	0.465
0.647	0.618	0.766	0.967	0.972	0.231	0.161	0.995	0.456	0.451
0.706	0.615	0.766	0.973	0.974	0.239	0.157	0.995	0.465	0.447
0.765	0.612	0.766	0.978	0.978	0.246	0.153	0.995	0.47	0.433
0.824	0.61	0.766	0.984	0.982	0.253	0.149	0.995	0.479	0.423
0.882	0.607	0.766	0.99	0.987	0.259	0.146	1.000	0.484	0.414
0.941	0.604	0.766	0.995	0.994	0.265	0.142	1.000	0.488	0.405
1.000	0.602	0.766	1.000	1.000	0.271	0.139	1.000	0.488	0.4

In contrast to what had been described above concerning the influence of *K*_2_ spring constant, an increase in the value of the spring *K*_1_ leads to the shifting of the third natural frequency while the second natural frequency remains stationary and the first one is very slightly reduced.
